# Climate change and hyponatremia‐related hospital admissions in people with focal epilepsy exposed to carbamazepine or its derivatives

**DOI:** 10.1111/epi.18584

**Published:** 2025-08-02

**Authors:** Francesco Fortunato, Francesco D'Amico, Anny Votano, Ilaria Sammarra, Environmental Climate Observatory Group – CNR‐ISAC Lamezia Terme, Claudia R. Calidonna, Ivano Ammoscato, Teresa Lo Feudo, Daniel Gullì, Luana Malacaria, Giorgia De Benedetto, Salvatore Sinopoli, Michele Trimboli, Medine I. Gulcebi, James D. Mills, Simona Balestrini, Sanjay M. Sisodiya, Antonio Gambardella

**Affiliations:** ^1^ Department of Medical and Surgical Sciences, Institute of Neurology Magna Graecia University Catanzaro Italy; ^2^ Department of Clinical and Experimental Epilepsy, UCL Queen Square Institute of Neurology University College London London UK; ^3^ Chalfont Centre for Epilepsy Chalfont‐St‐Peter UK; ^4^ Institute of Atmospheric Sciences and Climate National Research Council of Italy Lamezia Terme Italy; ^5^ Department of Biology, Ecology and Earth Sciences University of Calabria Rende Italy; ^6^ Department of Medical Pharmacology Marmara University School of Medicine Istanbul Turkey; ^7^ Department of Neuroscience and Medical Genetics Meyer Children's Hospital IRCSS Florence Italy; ^8^ University of Florence Florence Italy

**Keywords:** antiseizure medication, carbamazepine, climate change, focal epilepsy, hyponatremia

## Abstract

**Objective:**

To estimate the proportion of individuals with focal epilepsy treated with at least one among carbamazepine (CBZ), oxcarbazepine (OXC), or eslicarbazepine (ESL), who were hospitalized due to hyponatremia‐related symptoms in 2024, and to test the hypothesis that there is an association with climatic variables.

**Methods:**

We undertook a prospective study in which people with focal epilepsy treated with at least one of the target drugs and at least one attendance in 2024 formed the study cohort. Individuals who were admitted or seen as outpatients for hyponatremia in 2024 were considered cases and the rest considered controls. Climate analysis was performed in Lamezia Terme, Calabria, Italy.

**Results:**

Seventeen of the entire cohort of 105 (16.2%) had hyponatremia‐related hospitalizations. Older age (odds ratio [OR] = 1.07, 95% confidence interval [CI] = 1.03–1.12; *p* = .001) and exposure to OXC/ESL compared to CBZ (OR = 4.15, 95% CI = 1.20–14.32; *p* = .02) emerged as significant predictors of the events. Thirteen of the 17 cases (76.5%) currently reside on the Calabria coastline. Twelve of 17 events (70.6%) occurred between June and August. Among climatic variables, heatwaves (OR = 4.87, 95% CI = 1.75–13.50; *p* = .002) and tropical nights (night‐temperature ≥20°C) (OR = 2.72, 95% CI = 1.02–7.27; *p* = .046) were the most significant predictors of the events. Forecasting models based on 10 consecutive days of recordings prior to the events revealed trends of rising temperatures preceding the events.

**Significance:**

We report a high rate of hyponatremia‐related hospitalizations among people with epilepsy occurring predominantly during summer. Climate change–related events, such as heatwaves and tropical nights, may trigger hyponatremia symptoms. Climate–regional vulnerability should therefore also be considered when selecting antiseizure medications and when counseling patients. We encourage interdisciplinary collaboration between clinicians and climate scientists in this emerging critical area.

**Key points**

- We report the impact of climatic variables on hyponatremia‐related hospitalizations in a well‐phenotyped cohort of people with focal epilepsy exposed to carbamazepine or its derivatives.
- We identified heatwaves and tropical nights—defined as nights with temperatures persistently at or greater than 20°C—as the strongest predictors of hyponatremia‐related hospital admissions in Calabria, a climatically vulnerable region of southern Italy.
- This work represents a rare example of clinical research at the interface of epilepsy and climate science, illustrating the value of collaborative efforts between neurologists and climate experts.
- Regional climate vulnerability should be recognized as a new relevant factor to be considered when selecting, and counseling about, antiseizure medications.

## 1 INTRODUCTION

Anthropogenic climate change—characterized by global warming and an increased frequency of extreme weather events—constitutes a serious challenge for humanity.^1^ Individuals with neurological diseases, whose adaptive resilience may be compromised, are likely to be disproportionately affected.^2^, ^3^ People with epilepsy might be particularly vulnerable, as climatic variables have been associated with an increased frequency of seizures, potential alterations of antiseizure medication (ASM) efficacy, and a worsening of existing comorbidities.^4^, ^5^, ^6^ Thus, there is an urgent need for neurologists to engage constructively with the climate crisis by raising awareness, promoting mitigation strategies, and supporting further research initiatives,^7^ including into impacts upon, and consequences of, ASMs in the context of climate change.

Carbamazepine (CBZ) and its derivatives, oxcarbazepine (OXC) and eslicarbazepine (ESL), are among the most commonly prescribed ASMs.^8^ According to the latest (2023) report by the Italian Medicines Agency (AIFA), CBZ ranked as the third most prescribed ASM in Italy and the eighth most prescribed central nervous system drug in Calabria (Tables S1 and S2). Hyponatremia, defined as a serum sodium level below 135 mmol/L, with its associated symptoms, is a common consequence for people treated with these medications and may sometimes cause serious clinical outcomes.^9^ Hyponatremia^10^, ^11^, ^12^, ^13^, ^14^, ^15^, ^16^, ^17^ has been reported to affect up to 13.5% of individuals treated with CBZ,^11^ up to 29.9% of those treated with OXC,^11^ and between 14% and 20% of those treated with ESL.^17^ In particular, severe hyponatremia‐related symptoms^18^ such as behavioral disturbances, confusion, gait ataxia with falls, and increased seizure frequency, may be a significant cause of hospitalization or discontinuation of treatment with CBZ, OXC, or ESL.^17^, ^19^ For instance, a large Swedish study investigating drug‐induced hyponatremias found that CBZ was associated with the highest adjusted odds ratio (OR) for hospitalization (9.63, 95% confidence interval [CI] 6.18–15.33).^19^ Moreover, a recent cohort study reported that ESL‐induced hyponatremias caused severe symptoms in 6.1% of cases and resulted in treatment discontinuation in 6.2%.^17^ The mechanisms underlying hyponatremia are complex and multifactorial, with several concomitant medications^20^ and comorbidities potentially exacerbating both the symptoms and the risk of hospitalization.^9^, ^21^, ^22^ Notably, the global incidence of hyponatremia per 1000 person‐years has risen from 36.8% in 2007 to 58.5% in 2020 among older adults with epilepsy, many of whom have comorbidities and are on polypharmacy, which may act synergistically.^16^ Thus, in the context of an aging population and the rising prevalence of epilepsy among older adults, the avoidance of hyponatremia has become increasingly relevant.^16^

Several reports indicate that climate change may be a key factor in the increased risk of both all‐cause and drug‐related hyponatremias.^23^, ^24^, ^25^, ^26^ For example, a recent study demonstrated that any‐cause hyponatremia‐related hospitalizations—including for people taking ASMs (although the number of people with epilepsy was not specified)—increased rapidly at 24 h mean temperatures above 15°C, reaching 2.26 hospitalizations per million days at the highest recorded temperature of 25°C.^24^ To date, no research has explicitly explored the effect of climatic variables on individuals with epilepsy experiencing hyponatremia.

We have observed over the last few years increased numbers of people with epilepsy with hyponatremia‐related symptoms, some of whom have required hospitalization and urgent treatment in our Epilepsy Centre in Catanzaro (Calabria, Southern Italy). Calabria's narrow peninsular shape—culminating in the 32 km‐wide Catanzaro isthmus between the Tyrrhenian and Ionian coasts—renders it especially vulnerable to Mediterranean climate drivers.^27^ As one of the world's largest semi‐enclosed basins, the Mediterranean amplifies air‐mass transport^28^ and warming trends^29^; Calabria, in turn, experiences more frequent heatwaves and Saharan dust incursions,^30^ both of which heat air masses and elevate temperatures.

With these concerns in mind, we estimate the proportion of individuals with epilepsy treated with CBZ or its derivatives (“target ASMs”) hospitalized or seen as outpatient due to hyponatremia‐related symptoms from our center in 2024—the hottest year on record,^31^ and test the hypothesis that there is an association with climatic variables.

## 2 MATERIALS AND METHODS

### 2.1 Ethics statement

This research was approved by the relevant ethics committee (CET Calabria:21/Oct/2010‐ID:2021‐002637‐42) and conducted in accordance with the Helsinki Declaration and its later amendments. Written informed consent for research use of clinical data was obtained from individuals or legal guardians for those with intellectual disability.

### 2.2 Cohort selection

This prospective study was conducted at the Epilepsy Clinic in University “Magna Graecia” of Catanzaro. The study was conducted according to the STROBE (Strengthening the Reporting of Observational Studies in Epidemiology) guidelines (Data S1). Between January 1, 2024, and December 31, 2024, we consecutively enrolled adult individuals with focal epilepsy treated with target ASMs who received an outpatient visit or were admitted as inpatients to our neurology ward for symptoms related to hyponatremia. Specifically, the inclusion criteria were as follows: (1) age ≥18 years at the time of outpatient visit or hospitalization; (2) diagnosis of focal epilepsy according to the latest International League Against Epilepsy (ILAE) classification criteria^32^; (3) treatment regimen at the time of in‐person consultation or hospitalization included at least one among the target ASMs; (4) symptoms spontaneously reported by patients consistent with hyponatremia, such as behavioral disturbances, confusion, gait ataxia with falls, increased seizure frequency leading to an outpatient visit or hospitalization; (5) documented serum sodium level below 135 mmol/L, temporally associated with the aforementioned symptoms and with other potential causes reasonably excluded (Data S2); and (6) municipality of residence within the region of Calabria. Hyponatremia was classified as mild (serum sodium 130 to <135 mmol/L), moderate (120–129 mmol/L) and severe (<120 mmol/L). Three trained neurologists (F.F., A.V., and I.S.) with a special interest in epilepsy, collected the following information: sex; age at seizure onset; age at outpatient visit or hospitalization; air conditioning at patient's residence; seizure frequency at observation; epilepsy syndrome according to updated 2022 ILAE criteria; any structural cause of focal epilepsy as per brain magnetic resonance imaging (MRI); drug resistance as defined by the ILAE criteria^33^; ASMs at the time of the outpatient visit or hospitalization, including blood levels; concomitant medications with particular attention to drugs potentially related to hyponatremia such as diuretics, angiotensin receptor blockers (ARBs); angiotensin converting enzyme (ACE) inhibitors; proton pump inhibitors (PPIs) or anti‐depressants^21^; serum sodium levels; symptoms related to hyponatremia; and comorbidities with specific dietary regimens.

In addition, the Frailty Index (FI) was obtained for the study cohort. This 33‐item comprehensive scale has recently been recognized as a valuable tool for exploring multimorbidity in epilepsy.^34^ Further details on the single components of the scale are provided in Table S3.

### 2.3 Control cohort

We consider as a control cohort all adult individuals with focal epilepsy exposed to the target ASMs from our entire epilepsy outpatient list who attended at least one outpatient visit or were admitted as inpatients to our neurology ward in 2024, and neither had hyponatremia nor reported hyponatremia‐related symptoms. Further details on the control cohort may be found in Data S3.

### 2.4 Surface temperature and humidity measurements

Surface measurements of air temperature (T, °C) and relative humidity (RH, %) were recorded at the World Meteorological Organization – Global Atmosphere Watch (WMO/GAW) station of Lamezia Terme (LMT; Latitude: 38°52.605′ N; Longitude: 16°13.946′ E), Catanzaro, Calabria (Italy). Specifically, a Vaisala‐WXT520 weather station performed continuous measurements of temperature and RH on a per‐minute basis. For the purpose of this research, hourly‐aggregated data have been used.^35^ These data were further evaluated by a climate scientist (F.D.) to generate daily and monthly temperature and RH means, maxima, minima, ranges (max–min), standard deviations (SDs), and 5‐day moving averages, which are widely used in environmental studies to test the effects on human health.^36^ Additional information concerning these measurements can be found in Data S4 and in the glossary of climatic parameters, with their respective definitions provided in Table 1.

**TABLE 1:** Glossary of the climatic parameters.

Climatic variable	Definition	Measurement unit
Air daily temperature	Average air temperature measured over a 24‐h period	Celsius degrees (°C)
Air daily temperature SD	Standard deviation of the air temperature over a 24‐h period	Celsius degrees (°C)
Air daily minimum temperature	Lowest air temperature recorded during a 24‐h period	Celsius degrees (°C)
Air daily maximum temperature	Highest air temperature recorded during a 24‐h period	Celsius degrees (°C)
Air diurnal temperature range (DTR)	Difference between the daily maximum and minimum air temperatures	Celsius degrees (°C)
Daily RH	Average relative humidity measured over a 24‐h period	Percentage (%)
Daily RH SD	Standard deviation of relative humidity over a 24‐h period	Percentage (%)
Daily minimum RH	Lowest relative humidity recorded during a 24‐h period	Percentage (%)
Daily maximum RH	Highest relative humidity recorded during a 24‐h period	Percentage (%)
Diurnal RH range	Difference between the daily maximum and minimum relative humidity	Percentage (%)
Roll‐5T	5‐Day rolling (or moving) average of daily air temperature, smoothing short‐term fluctuations	Celsius degrees (°C)
Roll‐5T max	5‐Day rolling average of maximum temperatures	Celsius degrees (°C)
Roll‐5T min	5‐Day rolling average of minimum temperatures	Celsius degrees (°C)
Roll‐5T range	5‐Day rolling average of the difference between maximum and minimum temperatures	Celsius degrees (°C)
Roll‐5RH	5‐Day rolling (or moving) average of daily relative humidity, smoothing short‐term fluctuations in humidity	Celsius degrees (°C)
Roll‐5RH max	5‐Day rolling average of maximum relative humidity	Celsius degrees (°C)
Roll‐5RH min	5‐Day rolling average of minimum relative humidity	Celsius degrees (°C)
Roll‐5RH range	5‐Day rolling average of the difference between maximum and minimum relative humidity	Celsius degrees (°C)
Heath index	Please see Data S5	
Tropical nights	Nights during which air temperatures consistently exceeded 20°C	Celsius degrees (°C)
Heat waves	Please see Data S6	

Abbreviations: max, maximum; min, minimum; RH, relative humidity; SD, standard deviation.

**FIGURE 1:**
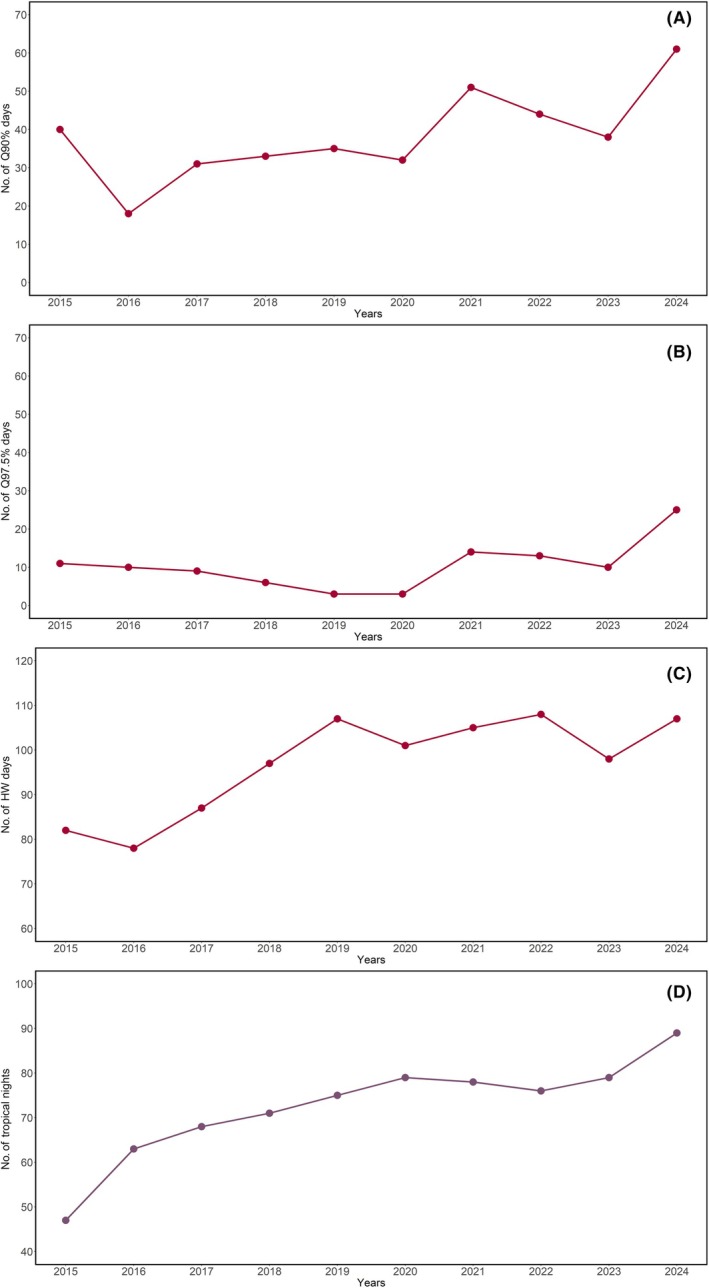
Number of heatwaves (HW) and tropical nights per year in Calabria (interval from 2015 to 2024). The x‐axis represents the years and the y‐axis the number of heatwave‐days, defined as: Air temperature equal or greater than 90th (A) and 97.5th percentiles (B) and maximum temperature exceeding by at least 5°C the multi‐year average (C). In (D) the x‐axis indicates the number of tropical nights, defined as nights with ambient temperatures at or exceeding 20°C. This analysis was performed at World Meteorological Organization – Global Atmosphere Watch (WMO/GAW) station of Lamezia Terme (LMT; Latitude: 38°52.605′ N; Longitude: 16°13.946′ E; Elevation: 6 meters above sea level).

Based on precise temperature and RH data, we calculated three derived metrics: (1) the heat index (HI), as an estimate of perceived temperature by integrating the effects of air temperature and RH (Data S5); (2) heatwaves, calculated with three different methods, defined in Data S6 and Figure 1A–C; and (3) tropical nights, defined as nights during which the minimum temperature does not drop below 20°C (Figure 1D).

### 2.5 Copernicus European Centre for Medium‐Range Weather Forecasts re‐analysis data

Copernicus European Centre for Medium‐Range Weather Forecasts (ECMWF) reanalysis five (ERA5) products^37^ were used to assess monthly temperature and RH across all municipalities of residence for the included cases (for additional information see Data S7).

### 2.6 Statistical analysis

Statistical analysis was performed using R software (v4.4.2). The normal distribution of each variable was assessed through Shapiro–Wilk test and visual inspection of histograms, Q‐Q plots, and box plots. Data were considered normally distributed for *p*‐value above .05. Homoscedasticity was evaluated with Levene's test. The chi‐square or Fisher's exact tests, as appropriate, for binary or categorical variables, and *t*‐test for continuous variables, were used for association tests. Multiple comparison correction was performed using false discovery rate (FDR) correction.

A multivariate logistic regression analysis with generalized linear models (GLMs) was conducted comparing cases to controls, using the following equation: events (i.e., outpatient visit or hospitalization due to symptomatic hyponatremia)~β₀ + Age (years) + Gender + Number of ASMs + OXC or ESL use. Temporal comparisons between the events and surface measurements and GLMs evaluating the association between each climatic variable and the occurrence of the events were built (for further details see Data S8).

## 3 RESULTS

### 3.1 Cohort description

In 2024, a total of 164 unique individuals with focal epilepsy who were treated with at least one of the targets ASMs attended at least one outpatient visit at our epilepsy clinic. Of these, 59 individuals (37.11%) were excluded from the analysis due to the absence of documented serum sodium measurements during the study year. Among the remaining 105 individuals, 17 (16.2%; (6 females; mean age 56.7 ± 16.4 years] required an outpatient visit or hospitalization due to symptoms associated with hyponatremia and were included in the case cohort. The remaining 88 individuals (83.8%) had documented serum sodium levels within the normal range and showed no symptoms of hyponatremia, thereby constituting the control cohort.

The demographic and clinical characteristics of our cohort are summarized in Table 2. None of the cases or controls followed any specific fluid restriction. Further details regarding individual cases are provided in Table S4. The exposure of our cases to the target ASMs was as follows: 7/17 (41.2%) on CBZ, 5/17 (29.4%) on ESL, and 5/17 (29.4%) on OXC. Concomitant ASMs in both cases and controls are presented in Figure S1. The mean age at epilepsy onset (Mann–Whitney test, *p* = .0001), mean age at observation (*t* test, *p* = .001), and the number of people who were following a low‐sodium diet due to arterial hypertension (chi‐square test, *p* = .001) for cases were significantly higher compared to the control cohort, and these differences survived after correction for multiple comparisons. There were no other significant differences between cases and controls.

**TABLE 2:** Demographic and clinical features of people with focal epilepsy and hyponatremia‐related symptoms and control cohort.

	Cases (*n* = 17)	Controls (*n* = 88)	*p*‐Value	*p*‐Value after Bonferroni correction (threshold < .002)
Gender, *n*° (female %)	6/17 (35.3%)	42/88 (47.7%)	.43^a^	ns
Age at epilepsy onset, years^b^	31.1 ± 18.9	13.5 ± 12.8	.0001^e^	Significant
Age at time of observation, years^b^	56.7 ± 16.4	41.3 ± 16.9	.001^c^	Significant
Seizure frequency at time of observation, *n*° (%)				
Daily	0/17	6/88 (6.8%)	.77^d^	ns
Weekly	4/17 (23.5%)	11/88 (12.5%)		
Monthly	4/17 (23.5%)	14/88 (15.9%)		
Yearly	3/17 (17.6%)	17/88 (19.3%)		
Seizure‐free	6/17 (35.3%)	30/88 (35.2%)		
NA	0/17	10/88 (11.4%)		
Structural epilepsy, *n*° (%)	8/17 (47%)	37/88 (42%)	.91^d^	ns
ASMs, *n*° (%)				
Monotherapy	4/17 (23.5%)	34/88 (38.6%)	.33^d^	ns
Bitherapy	6/17 (35.3%)	31/88 (35.2%)		
Polytherapy	7/17 (41.2%)	23/88 (26.1%)		
Drug resistance, *n*° (%)	8/17 (47%)	26/88 (29.5%)	.26^d^	ns
Type of target ASMs, *n*° (%)				
CBZ	7/17 (41.2%)	61/88 (69.3%)	.08^d^	ns
OXC	5/17 (29.4%)	12/88 (13.6%)		
ESL	5/17 (29.4%)	15/88 (17%)		
Daily dose of ASMs, mg/d^b^				
CBZ	1028.6 ± 335.2	858.2 ± 347.8	.14^e^	ns
OXC	1740 ± 684.1	1733.3 ± 745.1	.48^e^	ns
ESL	1520 ± 521.5	1250 ± 333.8	1^e^	ns
Serum levels of target ASMs, μg/mL^b^				
CBZ	9.5 ± 2.5	9 ± 2.2	.74^e^	ns
OXC	31 ± 1.4	16.1 ± 6.5	.03^e^	ns
ESL	22.5 ± 7.1	16.3 ± 8.4	.57^e^	ns
Serum levels of target ASMs out of the lab reference range, *n*° (%)	2/17 (11.8%)	4/88 (4.5%)	.18^a^	ns
33‐item Frailty Index^b^	.19 ± .16	.18 ± .13	.89^e^	ns
33‐item Frailty Index ≥0.20, *n*° %	8/17 (47%)	34/88 (38.6%)	.73^d^	ns
33‐item Frailty Index ≥0.25, *n*° %	5/17 (29.4%)	21/88 (23.9%)	.88^d^	ns
Air conditioning at home residence, *n*° (%)	5/17 (29.4%)	40/88 (45.6%)	.22^d^	ns
Current dietary regimens associated with medical comorbidities				
Low‐sodium diet related to arterial hypertension	7/17 (41.2%)	12/88 (13.6%)	.001^d^	Significant
Diabetes mellitus	1/17 (5.9%)	14/88 (15.9%)	1^a^	ns
Chronic kidney disease	0/17	14/88 (15.9%)	.77^a^	ns

Abbreviations: ASMs, antiseizure medications; CBZ, carbamazepine; ESL, eslicarbazepine; NA, not available; OXC, oxcarbazepine.

^a^ *p*‐Value after Fisher's exact test.

^b^ Data are expressed as mean ± standard deviation.

^c^ *p*‐Value after *t* test.

^d^ *p*‐Value after chi‐square test.

^e^ *p*‐Value after Mann–Whitney test.

A multivariate logistic regression analysis of demographic and clinical data with outpatient visits or hospitalization due to symptomatic hyponatremia as dependent variable is shown in Table 3.

**TABLE 3:** Multivariate logistic regression analysis of demographic and clinical data with outpatient visit or hospitalization due to symptomatic hyponatremia as dependent variable.

*N* = 105	Odds ratio	Lower 95% CI	Upper 95% CI	*p*‐Value
Age, years	1.07	1.03	1.12	.001**
Female gender	.69	.20	2.33	.54
Number of ASMs	1.58	.81	3.07	.17
OXC or ESL use^a^	4.15	1.20	14.32	.02*

Abbreviations: ASMs, antiseizure medications; CI, confidence interval; ESL, eslicarbazepine; OXC, oxcarbazepine.

^a^ Compared to CBZ exposure.

* *p*‐Value below .05.

** *p*‐Value below .01.

Older age at admission (OR = 1.07, 95% CI = 1.03–1.12; *p* = .001) and exposure to OXC/ESL compared to CBZ (OR = 4.15, 95% CI = 1.20–14.32; *p* = .02) emerged as significant predictors of outpatient visit or hospitalization due to symptomatic hyponatremia.

### 3.2 Climate analysis

#### 3.2.1 Temporal comparisons between the events and surface measurements

**FIGURE 2:**
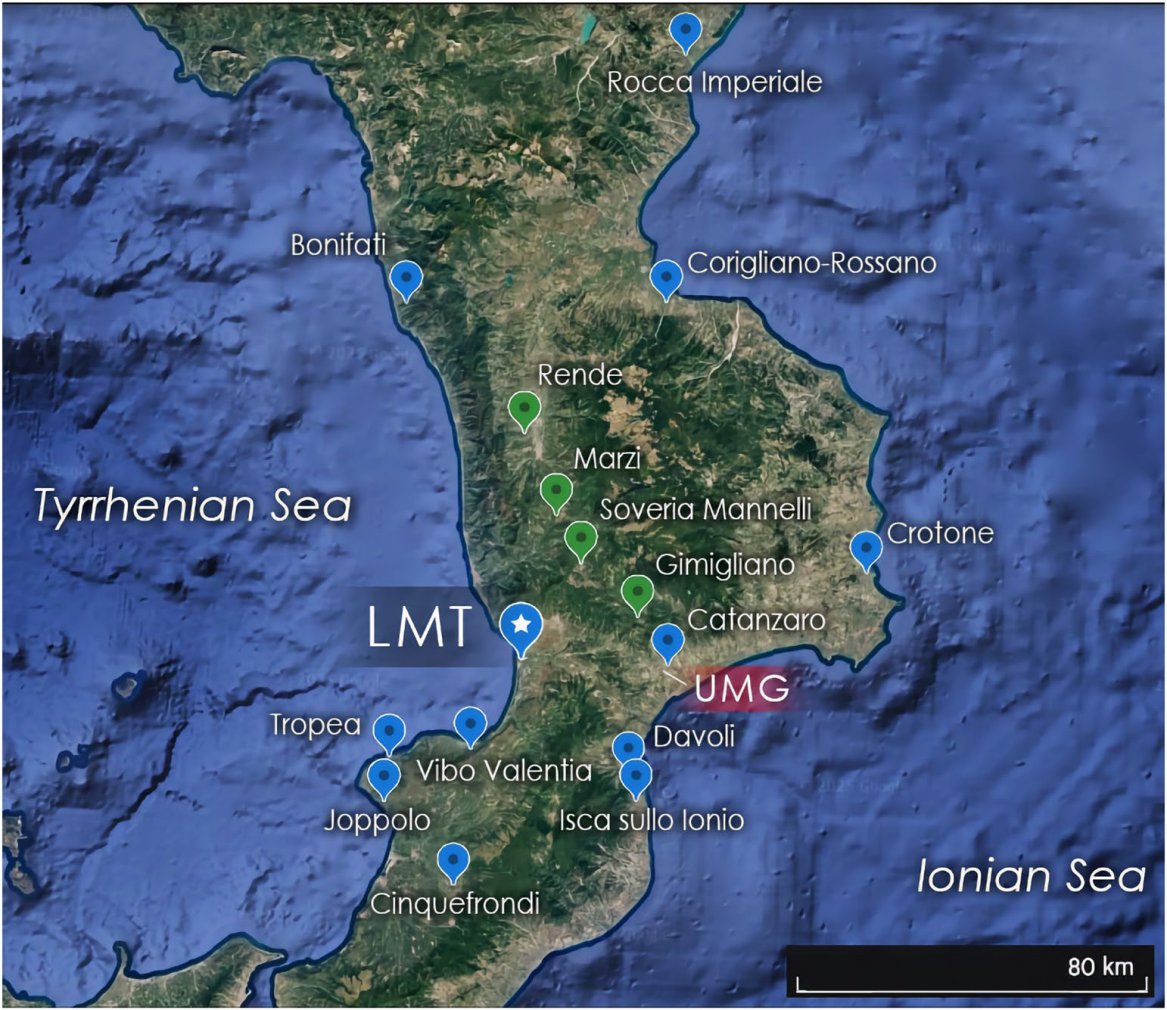
Municipalities of residence of the cases. Edited Google Earth map showing the municipalities of residence of the cases across the region of Calabria and the location of the Lamezia Terme (LMT) World Meteorological Organization – Global Atmosphere Watch (WMO/GAW) observation site. The boroughs of Corigliano and Rossano recently merged as “Corigliano‐Rossano”; therefore the median place between both boroughs is reported on this map. Blue marker with star: Lamezia Terme (LMT) World Meteorological Organization – Global Atmosphere Watch (WMO/GAW) observation site. UMG: Institute of Neurology, Magna Graecia University of Catanzaro. Blue markers indicate costal municipalities. Green markers indicate inland ones (this distinction is based on whether the municipalities have direct access to either the Tyrrhenian or Ionian Sea). The municipality of Cinquefrondi, located in the Gioia Tauro plain, is reported as coastal due to its low elevation compared to municipalities falling in the inland category.

Comprehensive geographical data (municipalities of residence of the cases) are provided in Figure 2. Notably 13 of 17 cases (76.5%) currently reside in the coastal areas of Calabria.

Climatic variables from the WMO/GAW monitoring station were validated against ERA5 reanalysis data at the station's coordinates and compared with ERA5‐derived values for each case's municipality of residence (for further details, see Figure S2, Table S5). Of interest, as shown in Figure 1, there was an increasing trend over the past decade in both average temperatures and the number of heatwaves (Figure 1A–C), as well as in the number of tropical nights (Figure 1D), with a peak in 2024—the hottest year on record.

The 17 events occurred on 16 dates during 2024. Twelve of 17 (70.6%) took place between June and August, with July being the month with the highest number (5/17), including two events that occurred on the same day (July 15, 2024).

**FIGURE 3:**
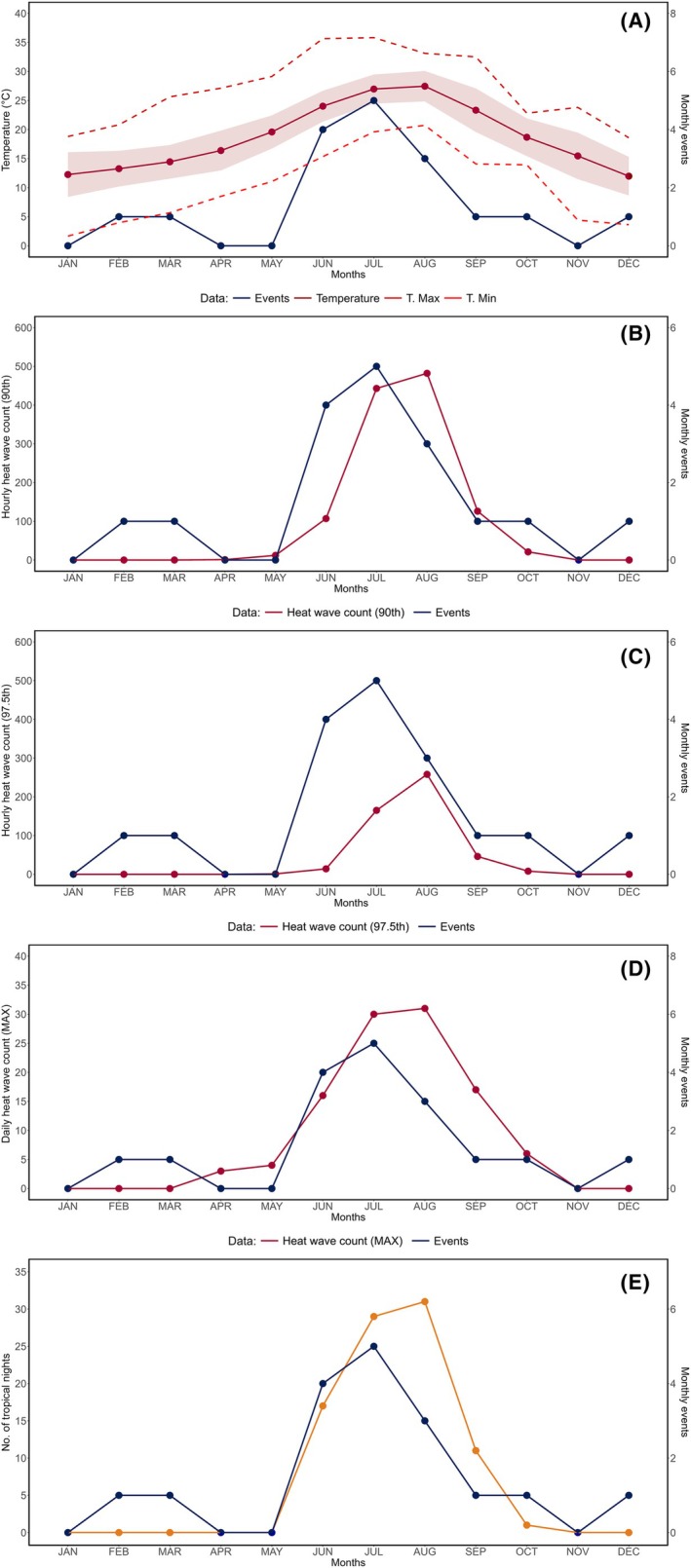
Events, temperature, and heatwaves. The figure illustrates the relationship, during the year 2024, between the number of events (*blue curve*) and the following climatic indicators: Temperature variation (A), heatwaves (B–D), and tropical nights (*orange curve*) (E). The x‐axis indicates the months of the year 2024, from January (left) to December (right). Heatwaves, shows in (B–D), were identified using temperature data from the preceding 10‐year period as a reference. Specifically, heatwaves are defined as: (B) the number of hours with temperatures exceeding the 90th percentile; (C) the number of hours exceeding the 97.5th percentile; and (D) the number of days with maximum temperatures exceeding the 10‐year average by at least 5°C.

Figure 3 illustrates an overview of the temporal trend of the events in relation to temperature measurements and heatwaves. Monthly temperatures measured at the LMT site correlated well with events (Figure 3A) under a linear regression (*R* ^2^ = .57; *p* = .005). There was a strong positive correlation between the occurrence of the events and the hours/days meeting heatwave criteria. Thus, the number of events/month yielded positive correlation with the sum (*R* ^2^ = .62; *p* = .002) and mean (*R* ^2^ = .62; *p* = .002) of hours exceeding the 90th percentile of temperature (Figure 3B); relevant results are also reported for the 97.5th percentile, both accounting for sum (*R* ^2^ = .44; *p* = .019) and mean (*R* ^2^ = .44; *p* = .019) (Figure 3C). We also found a significant correlation between monthly events and the number of days exceeding the average of the highest daily temperatures (Figure 3D) measured between 2015 and 2024 (*R* ^2^ = .68; *p* < .001) and with tropical nights (*R* ^2^ = .65; *p* < .001). Temporal trends of the events did not correlate with RH (Figure S3; *R* ^2^ = .02; *p* = .668).

Five of 17 events (29.4%) did not follow the clear trend of summer seasonality. However, when considering the overall distribution of climatic variables, a further link with them becomes apparent. As an example, a 62‐year‐old gentleman (EPICZ_014), who was receiving treatment with OXC 900 mg/day, was admitted due to confusion, lethargy, and syncope with moderate hyponatremia (sodium 122 mmol/L) in March 2024, on the most unseasonal day of the month, with the highest diurnal temperature range (14.5°C) and one of the 4 days of the year where the diurnal temperature range was at its maximum. A 27‐year‐old gentleman (EPICZ_017) who was taking ESL 2400 mg/day and valproate 1000 mg/day was admitted with fatigue and profound asthenia due to moderate hyponatremia in October 2024, on the day with the highest recorded RH maximum value of the month (98.9%) with a higher perceived temperature.

#### 3.2.2 Climatic variables as independent predictors of hospital admission for hyponatremia

**FIGURE 4:**
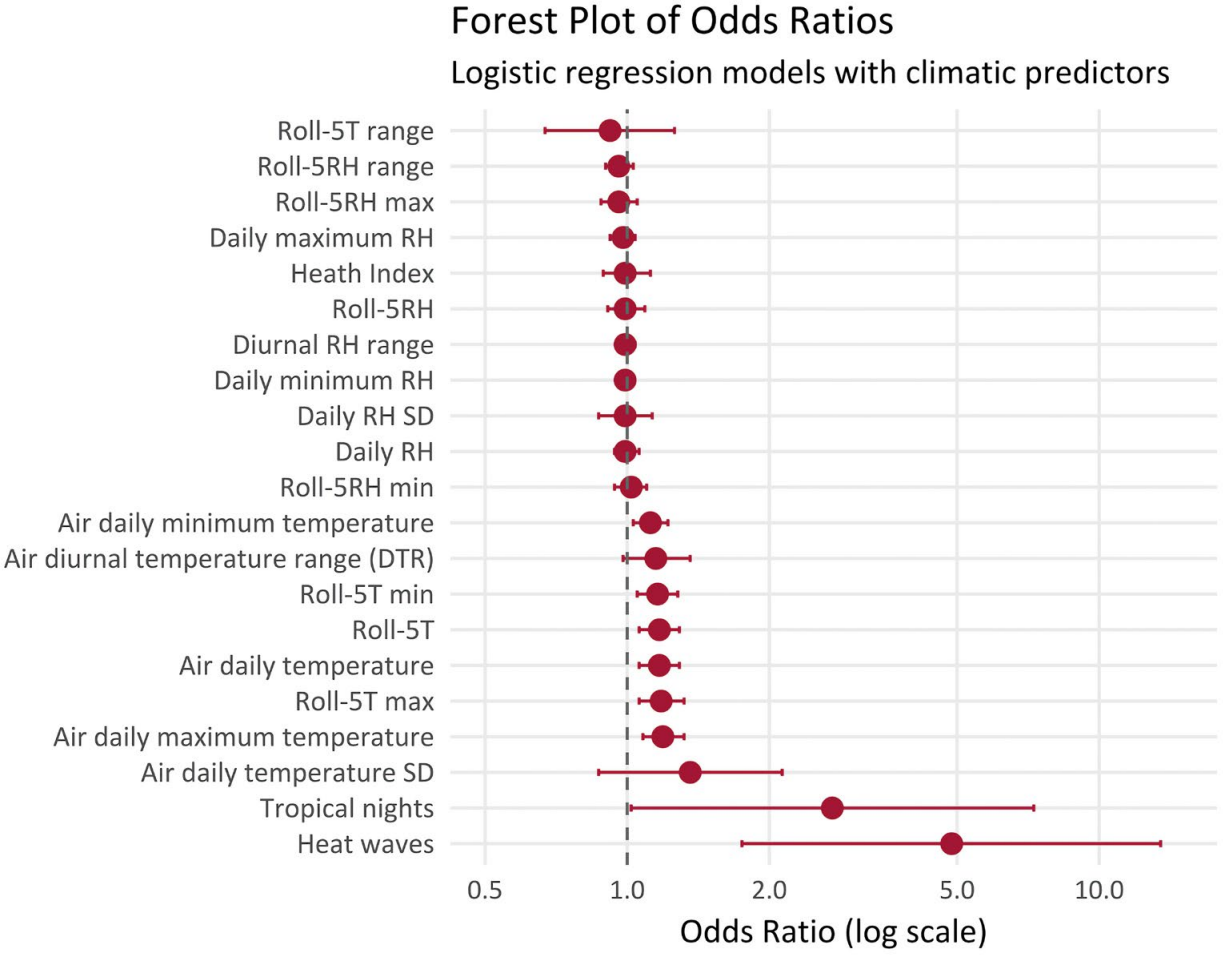
Forest plot of odds ratios (logistic regression models). The x axis shows the odds ratios (red circle) with the 95% CI (red bars). The y axis shows climatic variables. Roll‐5RH max, maximum value within the 5‐day rolling average of relative humidity; Roll‐5RH min, minimum value within the 5‐day rolling average of daily relative humidity; Roll‐5RH range, range value within the 5‐day rolling average of daily relative humidity; Roll‐5RH, 5‐day rolling average of relative humidity; Roll‐5T max, maximum value within the 5‐day rolling average of daily air temperature; Roll‐5T range, range value within the 5‐day rolling average of daily air temperature; Roll‐5T, 5‐day rolling average of daily air temperature; Roll‐5T min, minimum value within the 5‐day rolling average of daily air temperature; SD, standard deviation.

To explore why a person may have developed symptoms related to hyponatremia on a specific day, we built individual GLMs with climatic variables as predictors (Figure 4; see Table S6 for details). Notably, air daily temperature (OR = 1.17, 95% CI: 1.06–1.29; *p* = .002, *q*‐value = .008) and its extremes—both minimum (OR = 1.12, 95% CI: 1.03–1.22; *p* = .010, *q*‐value = .03) and maximum (OR = 1.19, 95% CI: 1.08–1.32; *p* = .0007, *q*‐value = .008) demonstrated significant associations with the events after FDR correction. In addition, markers of thermal stress and climate change, such as tropical nights and heatwaves, revealed pronounced risks, particularly heatwaves, which yielded the highest OR (4.87, 95% CI: 1.75–13.50; *p* = .002, *q*‐value = .008]. Variables related to RH did not reach statistical significance.

#### 3.2.3 Evaluation of possible patterns in the occurrence of events

The evaluated climatic parameters were also analyzed by considering 10 continuous days, including the day of the event, to test the possibility of regular patterns prior to the events themselves. These occurrences were tested for temperature and RH (Table S7), their 5‐day moving average (Table S8), and their range (maximum minus minimum; Table S9). We observed different behaviors for each parameter, temperature means yielding statistically significant linear regressions 7 times (43.75%), 5‐day averages 10 times (57.82%), and ranges once (6.25%). Relative humidity yielded diverse results for daily means (2 times, 11.76%), 5‐day averages (6 times, 35.29%), and ranges (2 times, 11.76%). From these results, we can infer that the majority of events were preceded by days characterized by linear, mostly positive temperature trends.

## 4 DISCUSSION

During the year 2024, we identified 17 of 105 (16.2%) adult patients with focal epilepsy, treated with at least one among the target ASMs, who required an outpatient visit or hospitalization due to symptoms that turned out to be related to hyponatremia. This proportion is unusual compared to previous reports of severe hyponatremias or symptomatic cases.^11^, ^15^, ^23^ For instance, Berghuis et al.^13^ reported admissions related to hyponatremia in about 3% of individuals with epilepsy. In a cohort of 560 people with epilepsy, clinically significant hyponatremia was reported in 6%.^14^ Our cases who were hospitalized were older, more frequently exposed to OXC/ESL, and more likely to have drug resistance. Both age at observation and exposure to OXC/ESL emerged as significant independent predictors of admissions due to hyponatremia. These findings are not unexpected, as several studies have previously reported that older age, exposure to OXC/ESL rather than CBZ, and polytherapy are associated with a higher risk of hyponatremia.^11^, ^12^, ^13^, ^14^, ^23^

The key novel finding was the clear seasonal trend in the occurrence of the events, with 70.6% taking place between June and August—that is, during the period accepted by the climate science community as “summer.” Moreover, 76.5% of the cases lived in coastal areas of central Calabria, which appears to be more vulnerable to climatic events compared with inland municipalities. Even during hospitalizations occurring outside the summer, related weather measures were unseasonal, often at extremes compared to long‐term local averages. For instance, patient EPICZ_014 was admitted on one of the rare days on which the temperature range was exceptionally high. Consequently, we suggest that unseasonal events—whose frequency is increasing due to climate change—may independently elevate the risk of hyponatremia‐related symptoms and admissions, even without a heatwave.

We further conducted a detailed climate analysis, revealing a significant positive correlation between the occurrence of the events and temperature‐derived variables; however, no such correlation was observed for RH. Our models, with the events as the dependent variable and each climatic variable as a predictor, indicated the highest OR for heatwaves (OR = 4.87, 95% CI = 1.75–13.50), followed by tropical nights (OR = 2.72, 95% CI = 1.02–7.27). That these measures—heatwaves and tropical nights—were more strongly associated with the events, is concerning as they will become more frequent due to climate change. We therefore hypothesize that persistently higher mean temperatures, in conjunction with tropical nights, may trigger drug‐induced hyponatremia symptoms in individuals who have additional risk factors such as older age or polytherapy.

A recent study highlighted heat‐related all‐cause hyponatremia as a significant medical issue, particularly among older, more frail people.^25^ We hypothesized that the comprehensive 33‐item FI would be higher in our cases compared to controls; however, the results did not support our hypothesis: 9 of 17 cases (52.9%) had FI <.20. Although our data might be underpowered due to small case group, we find these results intriguing, as it may be that climatic variables may precipitate hyponatremia even in non‐frail individuals. It is important to note that most of our cases did not have severe hyponatremia (13/17 moderate and 1/17 mild), but they developed symptoms prompting neurological consultation. Thus, we suggest that climate change–related events may precipitate hyponatremia‐related symptoms even in case with hyponatremia in mild–moderate range, increasing the proportion of people on these drugs who may be vulnerable, possibly because hyponatremia becomes less tolerable during unseasonal weather.

We consider our study topical for several reasons. First, we provided empirical data based on a simple clinical observation—a rare example of clinical research in the climate–epilepsy area and an example of how the community can gather information by working with climate experts. We hope that this robust interdisciplinary partnership will encourage the scientific community to pursue further collaborative research in this increasingly critical area. Thus, monitoring of climatic variables, as is already a widespread practice, may also serve as an effective warning system for health care providers and patients, facilitating the implementation of mitigation strategies. This is particularly relevant given the high prevalence of focal epilepsy and the prescription burden of CBZ/OXC/ESL. Second, regional climate vulnerability may also need to be considered when selecting those ASMs. Thus, promoting awareness between people with epilepsy, for example, advising individuals who live in areas like Calabria, and monitoring serum sodium levels more carefully, may be important.

Our study has several limitations. The development of symptomatic hyponatremia is complex and multifactorial, and it is unlikely that hospital admissions can be explained solely by climatic variables. For example, we found that a lower, although not statistically significant, proportion of cases had air conditioning in their residences compared to the control cohort. Indeed, fluctuations in ambient temperature could also affect the stability and efficacy of the ASMs, which typically require storage under stable and cool conditions.^38^ Moreover, it is reasonably to speculate that sodium and water intake, as well as specific dietary regimens related to the ongoing comorbidities, may have influenced the incidence of hyponatremia. Specifically, the number of people following a specific low‐sodium diet due to arterial hypertension was higher in our cases compared to control cohort. It is worth noting that, although there has been a downward trend in habitual salt intake in Italy overall—including in the region of Calabria—the average daily salt consumption among Italian adults still exceeds the levels recommended by the World Health Organization (WHO).^39^, ^40^ Some of these factors—such as access to air conditioning or adherence to specific dietary behaviors—are potentially modifiable and could help mitigate the impact of heatwaves on vulnerable patients.

Other additional factors, such as patient behavior and willingness and ability to attend hospital, have not been considered. Moreover, polypharmacy may play a significant role in the onset of hyponatremia^20^, ^41^: 7/17 (41%) cases were receiving ARBs, ACE inhibitors, or PPIs in combination with the target drugs; pharmacodynamic interactions between ASMs may have contributed. We also acknowledge that although a clinically appropriate diagnostic flow was applied to each individual as needed, the causative association between symptoms related to admissions and hyponatremia might not be absolute. We restricted our analysis to individuals with focal epilepsy attending our Epilepsy Centre. Some symptomatic cases may have presented to other centers in Calabria. In addition, we excluded cases who did have serum sodium measurements and those who did not attend our center in 2024, thereby omitting asymptomatic hyponatremias. Finally, we did not include people with developmental and epileptic encephalopathies, who may be unable to express their symptoms and may therefore be less likely to present to hospital, yet represent a particularly vulnerable group. For all these reasons, our findings cannot serve as an epidemiological report, but it may still only be capturing only the tip of the iceberg. The true burden of iatrogenic hyponatremia may be substantially higher than we have observed.

Our research highlights serious challenges for people with epilepsy and health care systems in vulnerable coastal regions such as Calabria.^42^ Our concerns are further amplified by the Italian government's scant commitment to climate change and sustainability issues, which has resulted in Italy's ranking on the Climate Change Performance Index declining from the 23rd rank in 2022 to the 43rd rank in 2025.^43^

In ancient Greece, the Temple of Apollo at Delphi bore the inscription μηδὲν ἄγαν, which translates as “nothing in excess”—now applicable to our climate. We believe that the historical wisdom encapsulated in this inscription is particularly pertinent to the findings of our study, which indicate that hospital admissions for hyponatremia tend to coincide with weather extremes, thereby underscoring the impact of such conditions on health. Most importantly, μηδὲν ἄγαν—we must address the fundamental causes of anthropogenic climate change: managing its consequences will be complicated, expensive, and may not always be possible.

## AUTHOR CONTRIBUTIONS

**Francesco Fortunato:** Writing – original draft preparation, conceptualization, data curation, formal analysis, investigation, methodology, and supervision. **Francesco D'Amico:** Data curation, formal analysis, investigation, methodology, and writing – original draft preparation. **Anny Votano:** Data curation, formal analysis, and investigation. **Ilaria Sammarra:** Data curation, formal analysis, and methodology. **Michele Trimboli:** Data curation and investigation. **Medine I. Gulcebi:** Data curation, methodology, supervision, writing – review & editing, and formal analysis. **James D. Mills:** Data curation, methodology, supervision, writing – review & editing, and formal analysis. **Simona Balestrini:** Data curation, methodology, supervision, writing – review & editing, and formal analysis. **Sanjay M. Sisodiya:** conceptualization, data curation, investigation, methodology, supervision, and writing – review and editing. **Antonio Gambardella:** conceptualization, data curation, investigation, methodology, supervision, and writing – review and editing.

## CONFLICT OF INTEREST STATEMENT

None of the authors has any conflict of interest to disclose. We confirm that we have read the Journal's position on issues involved in ethical publication and affirm that this report is consistent with those guidelines.

## ETHICAL PUBLICATION STATEMENT

This study has been approved by our institution's Ethics Committee and has been performed in accordance with the ethical standards laid down in the 1964 Declaration of Helsinki and its later amendments. We obtained patients' informed consent, and data were treated according to the European regulation GDPR n. 2016/679.

## Supporting information


Appendix S1

Table S4

## Data Availability

De‐identified data that support the findings of this study are available upon request to the corresponding author.

## APPENDIX A

Environmental Climate Observatory Group – CNR‐ISAC Lamezia Terme: Claudia R. Calidonna^4^: ORCID 0000‐0002‐8233‐0040; Ivano Ammoscato^4^: ORCID‐ID 0000‐0003‐3964‐7824; Teresa Lo Feudo^4^: ORCID‐ID 0000‐0002‐1916‐0552; Daniel Gullì^4^: ORCID‐ID 0000‐0002‐5263‐5134; Luana Malacaria^4^: ORCID‐ID 0009‐0003‐7321‐2805; Giorgia De Benedetto^4^: ORCID‐ID 0009‐0005‐8425‐8387; Salvatore Sinopoli^4^: ORCID‐ID 0000‐0003‐2262‐3607.
