# Supplementary material for: Climate change and hyponatremia‐related hospital admissions in people with focal epilepsy exposed to carbamazepine or its derivatives
Source: Epilepsia. 2025 Aug 2;66(12):4698–711. doi: 10.1111/epi.18584 (PMC12779323; doi:10.1111/epi.18584)
Supplement: Supplementary file 1 — Appendix S1 [file EPI-66-4698-s002.docx]

**Supplementary Materials**

1. Table S1: The 2023 report by the by Italian Medicines Agency (AIFA) on defined daily doses per 1,000 inhabitants per day for antiseizure medications^1^ in Italy
2. Table S2: The 2023 report by the by Italian Medicines Agency (AIFA) on defined daily doses per 1,000 inhabitants per day for central nervous system medications^1^ in Calabria
3. Table S3: 33-Item Frailty Index
4. Data S1: STROBE (Strengthening the Reporting of Observational Studies in Epidemiology) Checklist
5. Data S2: Diagnostic work-up and exclusion of alternative causes for cases
6. Data S3: Control cohort inclusion criteria
7. Data S4: Surface Parameter Measurements from the WMO/GAW Surface Station
8. Data S5: Heat Index calculation
9. Data S6: Heat waves data selection
10. Data S7: Copernicus European Centre for Medium-Range Weather Forecasts reanalysis data
11. Data S8: Statistical analysis
12. Table S4: Demographic and clinical features of the included cases
13. Figure S1: Concomitant anti-seizure medications of the cohort
14. Figure S2: Comparison between monthly surface measurements performed at the WMO/GAW site and ERA5-products
15. Table S5: Validation of climatic surface measurements and their ERA5 counterparts
16. Figure S3: Relative humidity and events
17. Table S6: Logistic regression analysis with the exact dates of ‘in-person consultation’ or hospitalization due to symptomatic hyponatremia as dependent variable and each climatic variables as predictor.
18. Table S7: Linear regression equations for temperature and relative humidity across the 10-day period preceding each event (from day -9 to day 0)
19. Table S8: Linear regression equations for 5-day moving averages of temperature and relative humidity across the 10-day period preceding each event (from day -9 to day 0)
20. Table S9: Linear regression equations for maximum and minimum ranges of temperature and relative humidity across the 10-day period preceding each event (from day -9 to day 0)

**Table S1: The 2023 report by the Italian Medicines Agency (AIFA) on defined daily doses per 1,000 inhabitants per day for antiseizure medications^1^ in Italy.**

| **Anti-seizure medications** | **DDD/1,000 inhabitants·day^§^** |
| --- | --- |
| Levetiracetam | 2.8 |
| Valproic acid | 2.7 |
| Carbamazepine | 1.2 |
| Lamotrigine | 0.9 |
| Oxcarbazepine | 0.6 |
| Lacosamide | 0.5 |
| Topiramate | 0.4 |
| Perampanel | 0.1 |
| Brivaracetam | 0.1 |
| Cannabidiol | 0.05 |

**Legend: §=** this metric expresses, on average, how many “standard adult daily doses” of a drug are consumed each day for every 1,000 people in the population

References:

1 Italian Medicines Agency (AIFA). L’uso dei farmaci in Italia. 2023. Available at: https://www.aifa.gov.it/uso-dei-farmaci-in-italia. Accessed 02-May-2205.

**Table S2:** The 2023 report by Italian Medicines Agency (AIFA) on defined daily doses per 1000 inhabitants per day for central nervous system medications (CNS)^1^ in Calabria* .

| **CNS medication** | **DDD/1,000 inhabitants·day^§^** |
| --- | --- |
| Venlafaxine | 3.3 |
| Duloxetine | 3 |
| Levodopa/benserazide | 2.7 |
| Citalopram | 2.5 |
| Selegiline | 1.5 |
| Fluoxetine | 1.5 |
| Carbamazepine | 1.2 |
| Mirtazapine | 1.1 |
| Lamotrigine | 1 |
| Trazodone | 0.9 |
| Oxcarbazepine | 0.8 |
| Paracetamol/codeine | 0.8 |
| Biperiden | 0.8 |
| Haloperidol | 0.8 |
| Topiramate | 0.7 |
| Levodopa/carbidopa | 0.7 |
| Fluvoxamine | 0.7 |
| Clomipramine | 0.6 |
| Lacosamide | 0.5 |
| Pramipexole | 0.5 |
| Gabapentin | 0.5 |
| Lidocaine | 0.4 |
| Tapentadol | 0.3 |
| Safinamide | 0.3 |
| Melevodopa/carbidopa | 0.3 |
| Bupropion | 0.3 |
| Rotigotine | 0.2 |
| Naloxone/oxycodone | 0.2 |
| Almotriptan | 0.2 |
| Rizatriptan | 0.2 |
| Rasagiline | 0.2 |
| Paracetamol/oxycodone | 0.2 |
| Tramadol | 0.2 |
| Pyridostigmine | 0.2 |
| Ropinirole | 0.2 |
| Sumatriptan | 0.1 |
| Buprenorphine | 0.1 |
| Quetiapine | 0.1 |
| Eletriptan | 0.1 |
| Aripiprazole | 0.1 |

**Legend:** *= Calabria, South of Italy §= this metric expresses, on average, how many “standard adult daily doses” of a drug are consumed each day for every 1,000 people in the population

References:

1 Italian Medicines Agency (AIFA). L’uso dei farmaci in Italia. 2023. Available at: https://www.aifa.gov.it/uso-dei-farmaci-in-italia. Accessed 02-May-2205.

**Table S3: 33-ITEM FRAILTY INDEX (FI)^1^**

| **33-ITEM FRAILTY INDEX** | **Present=1** | **Absent=0** |
| --- | --- | --- |
| Comorbidities |  |  |
| 1. Hypertension |  |  |
| 2. Diabetes mellitus |  |  |
| 3. Dyslipidemia |  |  |
| 4. Osteoporosis |  |  |
| 5. Peripheral vascular disease |  |  |
| 6. Thyroid disorders |  |  |
| 7. Rheumatological diseases |  |  |
| 8. Gastrointestinal disorders |  |  |
| 9. Chronic infections |  |  |
| 10. Liver disease |  |  |
| 11. Chronic kidney disease |  |  |
| 12. Hematological disorders |  |  |
| 13. Pulmonary disease |  |  |
| 14. Coronary Artery Disease |  |  |
| 15. Malignancy |  |  |
| 16. Headache |  |  |
| 17. Hypoacusis |  |  |
| Current signs and symptoms |  |  |
| 18. Chronic fatigue |  |  |
| 19. Edema of the lower extremities |  |  |
| 20. Unintentional weight loss (>4.5 kg in the last 6 months) |  |  |
| 21. Chronic pain |  |  |
| 22. Episodes of spatial and temporal disorientation (non-epileptic) |  |  |
| 23. Falls (non-epileptic) |  |  |
| 24. Vertigo (non-epileptic) |  |  |
| 25. Mood disorders |  |  |
| 26. Anxiety disorders |  |  |
| 27. Sleep disorders |  |  |
| Current disabilities |  |  |
| 28. Difficulties in climbing stairs |  |  |
| 29. Urinary incontinence |  |  |
| 30. Walking impairment (<400 m without aid or rest) |  |  |
| 31. Limitation in lifting/carrying groceries |  |  |
| 32. Working impairment |  |  |
| 33. Cognitive impairment |  |  |
| **TOTAL** | /33 | |
| **FRAILTY INDEX (range 0-1)** |  | |

**Legend:** two different cutoffs were used to define high FI values in our cohort: ≥ 0.20 and ≥ 0.25

*References:*

1. Cerulli Irelli E, Borioni MS, Morano A, Mazzeo A, Moro P, Orlando B, et al. Frailty as a comprehensive health measure beyond seizure control in patients with epilepsy: A cross-sectional study Epilepsia. 2024 Jun;65:1658-1667.

**Data S1: STROBE (Strengthening the Reporting of Observational Studies in Epidemiology) checklist**

|  | Item Description | Location (or reason for not reporting) |
| --- | --- | --- |
| **Title and abstract** |  |  |
| [1a. Indicate the study’s design](https:/resources.equator-network.org/guidelines/strobe/items/title-abstract-indicate-study-design.html) | Indicate the study’s design with a commonly used term in the title or the abstract. | Abstract (page 2) |
| [1b. Abstract](https:/resources.equator-network.org/guidelines/strobe/items/abstract.html) | Provide in the abstract an informative and balanced summary of what was done and what was found. | Abstract, methods |
| **Introduction** |  |  |
| [2. Background / rationale](https:/resources.equator-network.org/guidelines/strobe/items/background-rationale.html) | Explain the scientific background and rationale for the investigation being reported. | Introduction |
| [3. Objectives](https:/resources.equator-network.org/guidelines/strobe/items/objectives.html) | State specific objectives, including any prespecified hypotheses. | Introduction |
| **Methods** |  |  |
| [4. Study design](https:/resources.equator-network.org/guidelines/strobe/items/study-design.html) | Present key elements of study design early in the paper. | Methods, paragraph 2.2 ‘cohort selection’ |
| [5. Setting](https:/resources.equator-network.org/guidelines/strobe/items/setting.html) | Describe the setting, locations, and relevant dates, including periods of recruitment, exposure, follow-up, and data collection. | Methods, paragraph 2.2 ‘cohort selection’ and paragraph 2.3 ‘control cohort’ |
| [6a. Eligibility criteria](https:/resources.equator-network.org/guidelines/strobe/items/eligibility-criteria.html) | **Cohort study:** Give the eligibility criteria, and the sources and methods of selection of participants. Describe methods of follow-up. **Case-control study:** Give the eligibility criteria, and the sources and methods of case ascertainment and control selection. Give the rationale for the choice of cases and controls. **Cross-sectional study:** Give the eligibility criteria, and the sources and methods of selection of participants. | Methods, paragraph 2.2 ‘cohort selection’ and paragraph 2.3 ‘control cohort’. Additional info in data S1, S2 |
| [6b. Matching criteria](https:/resources.equator-network.org/guidelines/strobe/items/matching-criteria.html) | **Cohort study:** For matched studies, give matching criteria and number of exposed and unexposed. **Case-control study:** For matched studies, give matching criteria and the number of controls per case. | Not applicable |
| [7. Variables](https:/resources.equator-network.org/guidelines/strobe/items/variables.html) | Clearly define all outcomes, exposures, predictors, potential confounders, and effect modifiers. Give diagnostic criteria, if applicable. | Methods and Supplementary: DataS1, S2, S3, S4, S5, S6, S7 |
| [8. Data sources / measurement](https:/resources.equator-network.org/guidelines/strobe/items/data-sources-measurement.html) | For each variable of interest give sources of data and details of methods of assessment (measurement). Describe comparability of assessment methods if there is more than one group. | Methods: paragraphs 2.2, 2.3, 2.4 2.5 2.6 and Supplementary:  DataS1, S2, S3, S4, S5, S6, S7 |
| [9. Bias](https:/resources.equator-network.org/guidelines/strobe/items/bias.html) | Describe any efforts to address potential sources of bias. | Methods: paragraphs 2.2, 2.3, 2.4 2.5 2.6 and Supplementary:  DataS1, S2, S3, S4, S5, S6, S7 |
| [10. Study size](https:/resources.equator-network.org/guidelines/strobe/items/study-size.html) | Explain how the study size was arrived at. | Not applicable |
| [11. Quantitative variables](https:/resources.equator-network.org/guidelines/strobe/items/quantitative-variables.html) | Explain how quantitative variables were handled in the analyses. If applicable, describe which groupings were chosen, and why. | Statistical analysis and supplementary data S7 |
| [12a. Statistical methods](https:/resources.equator-network.org/guidelines/strobe/items/statistical-methods-description.html) | Describe all statistical methods, including those used to control for confounding. | Statistical analysis and supplementary data S7 |
| [12b. Statistical methods – subgroups and interactions](https:/resources.equator-network.org/guidelines/strobe/items/statistical-methods-subgroups-interactions.html) | Describe any methods used to examine subgroups and interactions. | Not applicable |
| [12c. Statistical methods – missing data](https:/resources.equator-network.org/guidelines/strobe/items/statistical-methods-missing-data.html) | Explain how missing data were addressed. | Methods: paragraphs 2.2, 2.3 and Supplementary DataS1, S2, S3, S4, S5, S6, S7 |
| [12di. Statistical methods – loss to follow-up](https:/resources.equator-network.org/guidelines/strobe/items/statistical-methods-loss-to-follow-up.html) | **Cohort study:** If applicable, describe how loss to follow-up was addressed. | Not applicable |
| [12dii. Statistical methods – matching cases and controls](https:/resources.equator-network.org/guidelines/strobe/items/statistical-methods-matching-cases-controls.html) | **Case-control study:** If applicable, explain how matching of cases and controls was addressed. | Not applicable |
| [12diii. Statistical methods – sampling strategy](https:/resources.equator-network.org/guidelines/strobe/items/statistical-methods-analytical-methods-sampling-strategy.html) | **Cross-sectional study:** If applicable, describe analytical methods taking account of sampling strategy. | Not applicable |
| [12e. Statistical methods – sensitivity analyses](https:/resources.equator-network.org/guidelines/strobe/items/statistical-methods-sensitivity-analyses.html) | Describe any sensitivity analyses. | Not applicable |
| **Results** |  |  |
| [13a. Participant numbers](https:/resources.equator-network.org/guidelines/strobe/items/participants-numbers.html) | Report the numbers of individuals at each stage of the study—e.g., numbers potentially eligible, examined for eligibility, confirmed eligible, included in the study, completing follow-up, and analysed; Consider use of a flow diagram. | Results paragraph 3.1 Cohort description and Table S5 |
| [13b. Participants – non-participation](https:/resources.equator-network.org/guidelines/strobe/items/participants-non-participation.html) | Give reasons for non-participation at each stage. | Not applicable |
| [13c. Participants – flow diagram](https:/resources.equator-network.org/guidelines/strobe/items/participants-flow-diagram.html) | Consider use of a flow diagram. | Not applicable |
| [14a. Descriptive data – participant characteristics](https:/resources.equator-network.org/guidelines/strobe/items/descriptive-data-participant-characteristics.html) | Give characteristics of study participants (e.g., demographic, clinical, social) and information on exposures and potential confounders. Present the information in a table. | Results paragraph 3.1 Cohort description and Table S5 |
| [14b. Descriptive data – missing data](https:/resources.equator-network.org/guidelines/strobe/items/descriptive-data-missing-data.html) | Indicate the number of participants with missing data for each variable of interest. | Results paragraph 3.1 Cohort description and Table S5 |
| [14c. Descriptive data – follow-up time](https:/resources.equator-network.org/guidelines/strobe/items/descriptive-data-follow-up-time.html) | **Cohort study:** Summarise follow-up time—e.g., average and total amount. | Not applicable |
| [15. Outcome data](https:/resources.equator-network.org/guidelines/strobe/items/outcome-data.html) | **Cohort study:** Report numbers of outcome events or summary measures over time. **Case-control study:** Report numbers in each exposure category, or summary measures of exposure. **Cross-sectional study:** Report numbers of outcome events or summary measures. | Results paragraph 3.1 and 3.2 |
| [16a. Main results](https:/resources.equator-network.org/guidelines/strobe/items/main-results.html) | Give unadjusted estimates and, if applicable, confounder-adjusted estimates and their precision (e.g., 95% confidence intervals). Make clear which confounders were adjusted for and why they were included. | Results paragraph 3.2.2 |
| [16b. Main results – category boundaries](https:/resources.equator-network.org/guidelines/strobe/items/main-results-category-boundaries.html) | Report category boundaries when continuous variables were categorised. | Not applicable |
| [16c. Main results – risk](https:/resources.equator-network.org/guidelines/strobe/items/main-results-risk.html) | If relevant, consider translating estimates of relative risk into absolute risk for a meaningful time period. | Not applicable |
| [17. Other analyses](https:/resources.equator-network.org/guidelines/strobe/items/other-analyses.html) | Report other analyses done—e.g., analyses of subgroups and interactions, and sensitivity analyses. | Not applicable |
| **Discussion** |  |  |
| [18. Key results](https:/resources.equator-network.org/guidelines/strobe/items/key-results.html) | Summarise key results with reference to study objectives. | Discussion |
| [19. Limitations](https:/resources.equator-network.org/guidelines/strobe/items/limitations.html) | Discuss limitations of the study, taking into account sources of potential bias or imprecision. Discuss both direction and magnitude of any potential bias. | Discussion |
| [20. Interpretation](https:/resources.equator-network.org/guidelines/strobe/items/interpretation.html) | Give a cautious overall interpretation considering objectives, limitations, multiplicity of analyses, results from similar studies, and other relevant evidence. | Discussion |
| [21. Generalisability](https:/resources.equator-network.org/guidelines/strobe/items/generalisability.html) | Discuss the generalisability (external validity) of the study results. | Discussion |
| **Other information** |  |  |
| [22. Funding](https:/resources.equator-network.org/guidelines/strobe/items/funding.html) | Give the source of funding and the role of the funders for the present study and, if applicable, for the original study on which the present article is based. | No funding required |

**Data S2: Diagnostic work-up and exclusion of alternative causes for cases**

We call ‘cases’ the individual with focal epilepsy treated with CBZ or OXC or ESL(‘target ASMs’) who received an outpatient visit or were admitted as inpatients to our neurology ward for symptoms related to hyponatremia. Specifically, the inclusion criteria were as follows: 1)age ≥18 years at the time of in-person consultation or hospitalization; 2)diagnosis of focal epilepsy according to the latest ILAE classification criteria; 3)treatment regimen at the time of in-person consultation or hospitalization included at least one among CBZ or OXC or ESL; 4)symptoms consistent with hyponatremia- such as behavioural disturbances, confusion, gait ataxia with falls, increased seizure frequency that were the cause of an outpatient visit or hospitalization; 4)documented serum sodium level below 135 mmol/L, temporally associated with the aforementioned symptoms and with other potential causes reasonably excluded ; 5)municipality of residence within the region of Calabria (South of Italy). Hyponatraemia was defined as follows: mild with serum sodium levels between 130 and below 135 mmol/L; moderate between 120 and 129 mmol/L; severe below 120 mmol/L.

For each case presenting with symptoms temporally associated with documented hyponatraemia, a comprehensive diagnostic protocol was undertaken to confirm the association and to exclude other potential causes. In particular we conducted the following diagnostic-flow: 1) Detailed medical history, including medication review as detailed in the main text; 2) neurological examination; 3) standard EEG with at least 20-minute recording; 4) brain MRI with dedicated protocol for epilepsy. Additionally, as our Epilepsy Centre maintains a close collaboration with the Nephrology Unit, we consulted with them to be more confident in the association between the symptoms and hyponatraemia.

**Data S3: Control cohort inclusion criteria**

Detailed inclusion criteria for individuals with focal epilepsy consecutively enrolled between first January 2024 to 31^th^ December 2024 were as follows: 1) age ≥ 18 years at the time of outpatient visit or hospitalization; 2) treated at time of observation with at least one among carbamazepine (CBZ) or oxcarbazepine (OXC) or eslicarbazepine (ESL); 3) diagnosis of focal epilepsy according to the latest International League Against Epilepsy (ILAE)^1^ recommendation criteria; 4) documented normal serum sodium level at time of in-person consultation or hospitalization (i.e. between 136 to 145 mmol/L). All people who did not fullfill all of the aforementioned criteria were excluded from the final analysis.

*References:*

1. Scheffer IE, Berkovic S, Capovilla G, Connolly MB, French J, Guilhoto L, et al. ILAE classification of the epilepsies: Position paper of the ILAE Commission for Classification and Terminology Epilepsia. 2017 Apr;58:512-521.

**Data S4: Surface Parameter Measurements from the WMO/GAW Surface Station**

The World Meteorological Organization – Global Atmosphere Watch (WMO/GAW) station of Lamezia Terme (code: LMT) is equipped with two Vaisala WXT520 (Vantaa, Finland) weather stations performing continuous measurements of key near-surface meteorological data. These instruments measure vital parameters for local climate studies, such as air pressure, rain accumulation, wind direction, wind speed, and hail. For the purpose of this research, air temperature (T, °C) and relative humidity (RH, %) have been used.

Both T and RH are measured by the PTU module of the WXT520. The principle of operation is based on two reference capacitors, and an advanced Resistor-Capacitor (RC) oscillator. The capacitance of internal sensors against the reference capacitors is subject to continuous measurements. Inside the transmitter, a microprocessor compensates for the temperature dependency of the relative humidity sensors. Air temperature is directly measured by a capacitive ceramic THERMOCAP sensor, while capacitive thin film polymer HUMICAP 180 sensor is used for humidity.

WXT520 weather stations perform their measurements at an elevation of 10 meters above ground level (AGL). Considering the elevation of the observatory compared to sea level, which is 6 meters, the total elevation is 16 meters above sea level (ASL). As per the constructor’s recommendations, the meteorological tower (mast) on top of which the WXT520 is placed is located nearby the observation site’s shelter and set up to avoid direct turbulence interference from objects such as trees and buildings. The WXT520 has an air temperature operating range of -52 to +60 °C, with a resolution of 0.1 °C and a precision of ± 0.3 °C; the relative humidity operating range is 0-100%, with a resolution of 0.1% and an accuracy of ± 3% in the 0-90% range, and ±5% in the 90-100% range.

During the calendar year 2024 (01 January to 31 December), the combined coverage of Vaisala WXT520 instruments at LMT with respect to continuous hourly data is 95.07%. Of the remaining 4.93% which is not covered by measurements, 2.17% of it is due to a single maintenance issue occurred on October 21^st^-30^th^, during which none of the weather stations were available.

The instrument’s output is on a per-minute basis. Following the same methodology used in climate and environmental studies, data are aggregated on an hourly basis.

**Data S5: Heat Index calculation**

The Heat Index (HI) was calculated to estimate the perceived temperature by integrating the effects of air temperature and relative humidity (RH). The calculation followed the algorithm developed by the U.S. National Weather Service (NWS)^1^, as described by Rothfusz, which is widely used in climatological and public health research.

Specifically, raw air temperature data (in degrees Celsius) were first converted to degrees Fahrenheit.

The Heat Index was then computed using the Rothfusz regression equation:

HI_F_= −42.379 + 2.04901523T + 10.14333127RH −0.22475541TRH −0.00683783T^2^− 0.05481717RH^2^ +0.00122874T^2^RH + 0.00085282TRH^2^ − 0.00000199T^2^RH^2^

Where:

- HI_F_ is the Heat Index in Fahrenheit,
- T is the ambient air temperature in Fahrenheit,
- RH is the relative humidity (as a percentage).

The final Heat Index was converted back to degrees C° using the inverse of the initial transformation:

HI_C_ = HI_F_−32/ 1.8

This approach provides a more physiologically meaningful estimate of heat stress than temperature alone in the context of climate change^2^.

References:

1. Haque F, Lampe FC, Hajat S, et al. Heat Index: An Alternative Indicator for Measuring the Impacts of Meteorological Factors on Diarrhoea in the Climate Change Era: A Time Series Study in Dhaka, Bangladesh. *Int J Environ Res Public Health*. 2024;21(11):1481. Published 2024 Nov 7. doi:10.3390/ijerph21111481
2. Zeppetello LRV, Raftery AE, Battisti DS. Probabilistic projections of increased heat stress driven by climate change. Commun Earth Environ. 2022;3(1):183. doi: 10.1038/s43247-022-00524-4. Epub 2022 Aug 25. PMID: 39421457; PMCID: PMC11485542.

**Data S6: Heatwave data selection**

Three data selection and processing methods were used in this study to compute the heat waves during the calendar year 2024.

In the first two methods^1^, we employed the 90th and 97.5th percentiles, respectively, calculated from all hourly mean air temperatures recorded at the Lamezia Terme site of the Global Atmosphere Watch (WMO/GAW) station, using data from the local WXT520 weather station (a total of 87,672 hourly observations from 2015 to 2024). Hourly 2024 temperature data equal or greater than these thresholds have been counted for each day.

The third method^2^ was applied as follows: for each day between 2015 and 2024, the maximum hourly value has been selected; consequently, the average of all daily 2015-2024 maxima has been calculated. A filter has been applied to 2024 data to verify the occurrence of hours exceeding by at least 5 °C the multi-year average. All days in 2024 with one or more hours satisfying the condition were flagged for further analysis.

References:

1. Schoetter R, Cattiaux J, Douville H. Changes of western European heat wave characteristics projected by the CMIP5 ensemble Climate Dynamics. 2015 2015/09/01;45:1601-1616.
2. Radinović D, Ćurić M. Criteria for heat and cold wave duration indexes Theoretical and Applied Climatology. 2012 2012/02/01;107:505-510.

**Data S7: Copernicus European Centre for Medium-Range Weather Forecasts reanalysis data**

Copernicus C3S (Climate Change Service) ECMWF (European Center Medium Weather Forecast) ERA5 (ECMWF re-analysis 5) data products were used in this study to evaluate surface temperature and relative humidity on a monthly basis in the region of Calabria. The analysis was also aimed at LMT’s coordinates to verify the agreement of these products with local surface measurements. ERA5-Land monthly averages of temperature have a native spatial resolution of 9 kilometers, and an effective resolution of 0.1x0.1 degrees of latitude and longitude. Relative humidity data have a resolution of 0.25x0.25 degrees. An iterative MATLAB (v. R2016a) algorithm set up by CNR-ISAC LMT parses through available data, selecting those with coordinates matching the locations used in this study. The same algorithm also performs quality assurance checks meant to filter out data that do not match the recommended quality standards.

Specifically, ERA5-Land products, which are meant to account for the evolution of land variables, were processed and evaluated. ERA5-Land is characterized by a long time series, which allows direct comparison from 1950 onwards; the spatial resolution of these products with respect to surface temperature, is 9 kilometres on a reduced Gaussian grid (TCo1279).

ERA5 products by Copernicus were processed by an algorithm set up and developed by CNR-ISAC Lamezia Terme. The algorithm parses through the dataset to select measurements linked to target coordinates, and quality assurance checks ensure that only data deemed valid for evaluation are considered. All data that are not deemed representative of the selected coordinates are excluded by the algorithm.

Relative humidity products are characterized by reduced spatial resolution (25x25 kilometres grids). Data and quality assurance checks performed by CNR-ISAC Lamezia Terme have covered all locations evaluated in this work, with the exception of Bonifati (province of Cosenza), as the gridded value for the municipality were not deemed representative of this coastal town. Several locations fall under the same grid sector, as they are located close to each other.

The general trend between 1979 and 2024 was calculated via a comparison between 1979-2000 and 2000-2024 average temperatures, which underline a general increase in temperatures across the European continent and beyond.

**Data S8: Statistical analysis**

For each recorded date on which cases accessed hospital services due to symptomatic hyponatraemia, the corresponding climatic parameters were examined on the exact date of the event, as well as using up to -10-day intervals preceding the event. The full list of climatic variables analysed is presented in Table 2. The resulting values were then compared against three reference distributions: 1) all daily data from the whole 2024; 2) daily data from the corresponding month; and 3) daily data from the corresponding season. In each case, the differences were assessed using a non-parametric Mann–Whitney test. A *p*-value <0.05 was considered significant after correction for multiple comparisons using the false discovery rate. Each season was defined using the leading convention in climate studies^1,2^:

1. December- January- February (DJF) for Winter
2. March-April-May (MAM) for Spring;
3. June-July-August (JJA) for Summer;
4. September-October-November (SON) for Autumn

Moreover, we tested whether each climatic variable, as measured on the exact date of the event could be considered an extreme outlier when compared with the distributions for the entire year 2024, the corresponding month, or the corresponding season. The outliers were defined as follows: the lower limit is defined as the first quartile minus 1.5 times the interquartile range (IQR), and the upper limit as the third quartile plus 1.5 times the IQR.

References:

1) Giorgi F, Bi X, Pal JS. Mean, interannual variability and trends in a regional climate change experiment over Europe. I. Present-day climate (1961–1990) Climate Dynamics. 2004 2004/06/01;22:733-756.

2) Xu W, Li Q, Jones P, Wang XL, Trewin B, Yang S, et al. A new integrated and homogenized global monthly land surface air temperature dataset for the period since 1900 Climate Dynamics. 2018 2018/04/01;50:2513-2536.

**Table S4: Demographic and clinical features of the included cases**

Please see the attached excel spreadsheet called ‘TableS4’.

**Figure S1: Concomitant anti-seizure medications of the cohort**

**Legend:** **cases:** the 17 individuals with focal epilepsy who were admitted at hospital due to symptoms related to hyponatremia; **controls:** the 88 controls with focal epilepsy who did not manifest hyponatremia; **PB**= phenobarbital; **LEV**= levetiracetam; **VPA**= valproic acid; **CLB**= clobazam; **BRIVA**= brivaracetam; **CNB**= cenobamate; **TPM**= topiramate; **LAC**= lacosamide; **PER**= perampanel; **ZNS**= zonisamide; **ACZ**= acetazolamide; **LTG**= lamotrigine; **VGA**= vigabatrin

**Figure S2: Comparison between monthly surface measurements performed at the WMO/GAW site and ERA5-products**


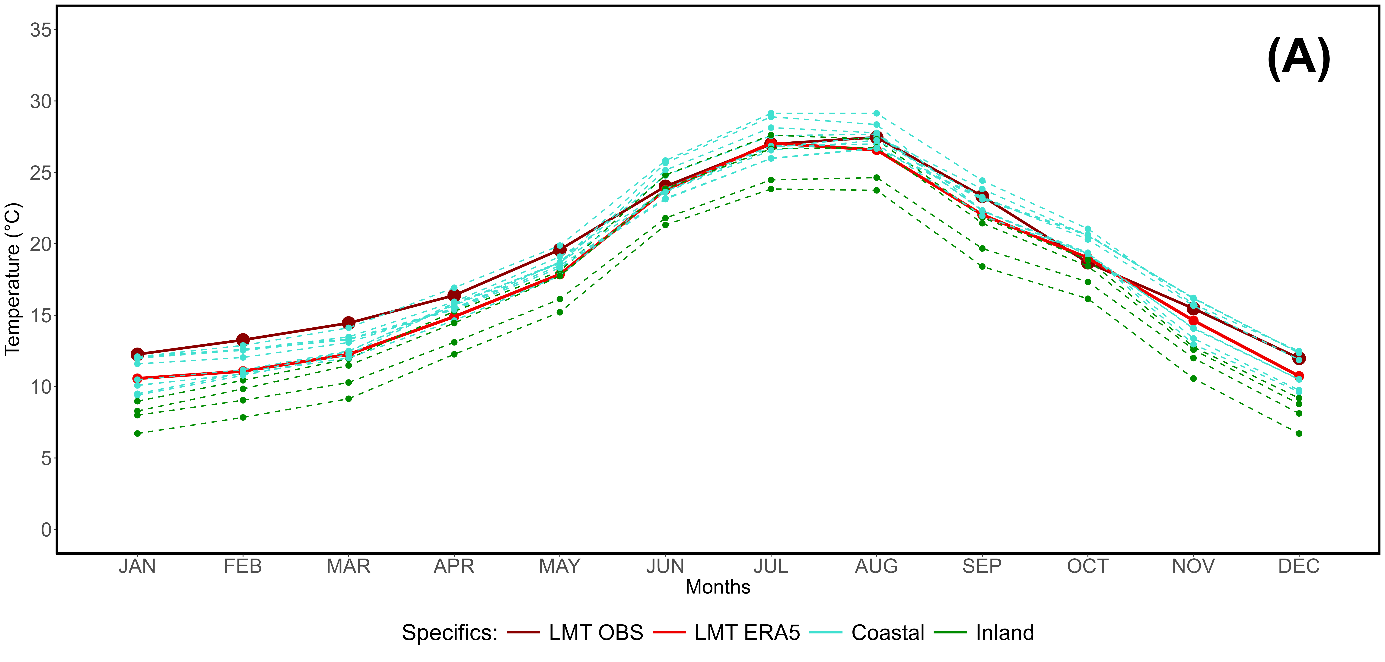


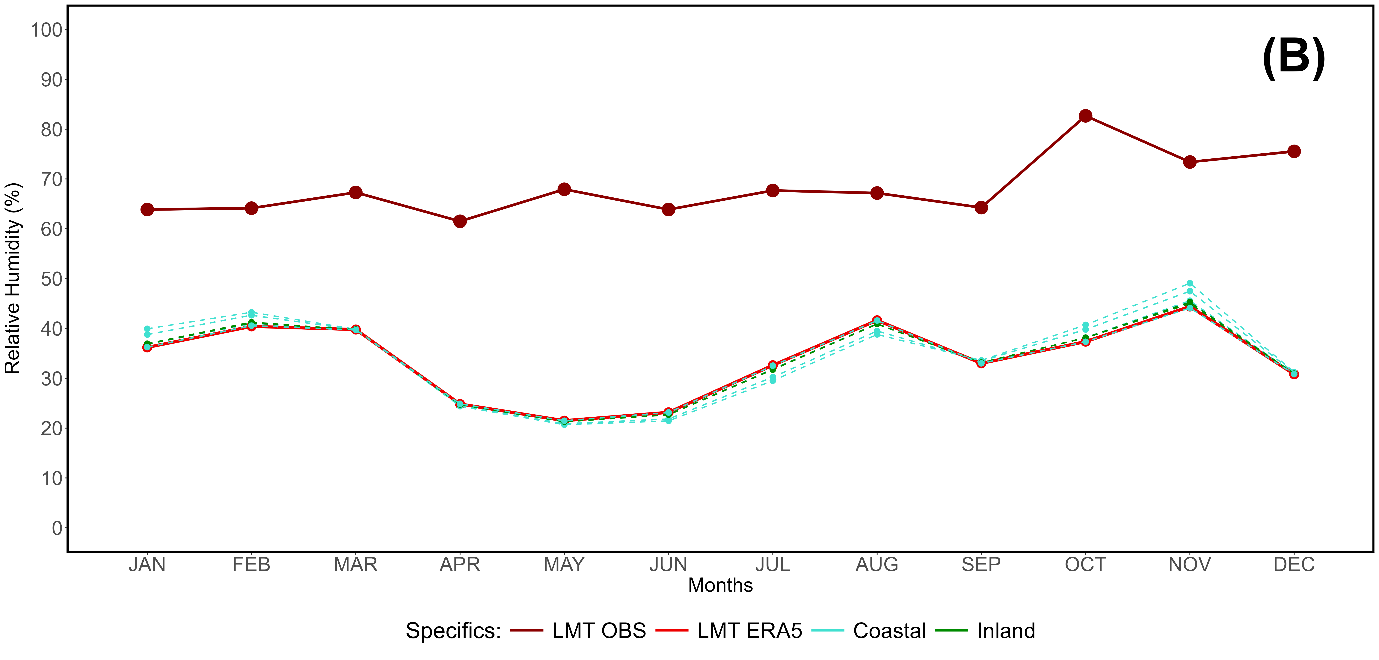


**Legend:** Comparison between monthly surface measurements performed at the WMO/GAW site (LMT OBS) and ECMWF products (LMT ERA5, Coastal, Inland) of temperature (A) and relative humidity (B).

**Table S5: Validation of climatic surface measurements and their ERA5 counterparts**

| Location | Type | Temperature (°C) | | | | Relative Humidity (%) | | | |
| --- | --- | --- | --- | --- | --- | --- | --- | --- | --- |
|  |  | int | m | R^2^ | *p*-value | int | m | R^2^ | *p*-value |
| Lamezia Terme | Observatory | -2.74 | 1.09 | 0.99 | < 0.001 | 9.12 | 0.36 | 0.08 | 0.36 |
| Bonifati | Coastal | 0.42 | 0.96 | 0.97 | < 0.001 | NA | NA | NA | NA |
| Catanzaro | Coastal | -3.56 | 1.15 | 0.99 | < 0.001 | 6.80 | 0.40 | 0.10 | 0.33 |
| Cinquefrondi | Coastal | -0.16 | 1.01 | 0.98 | < 0.001 | 9.54 | 0.36 | 0.08 | 0.37 |
| Corigliano | Coastal | -5.52 | 1.22 | 0.99 | < 0.001 | 2.29 | 0.47 | 0.11 | 0.29 |
| Crotone | Coastal | -1.71 | 1.14 | 0.99 | < 0.001 | 6.44 | 0.40 | 0.10 | 0.32 |
| Davoli | Coastal | -3.47 | 1.12 | 0.99 | < 0.001 | 6.80 | 0.40 | 0.10 | 0.33 |
| Gimigliano | Inland | -5.52 | 1.22 | 0.99 | < 0.001 | 6.80 | 0.40 | 0.10 | 0.33 |
| Isca | Coastal | -3.47 | 1.12 | 0.99 | < 0.001 | 6.80 | 0.40 | 0.10 | 0.33 |
| Joppolo | Coastal | 0.42 | 0.96 | 0.97 | < 0.001 | 9.54 | 0.36 | 0.08 | 0.37 |
| Marzi | Inland | -5.10 | 1.10 | 0.99 | < 0.001 | 7.23 | 0.39 | 0.09 | 0.34 |
| Rende | Inland | -5.78 | 1.20 | 0.99 | < 0.001 | 6.80 | 0.40 | 0.10 | 0.33 |
| Rocca | Coastal | -5.83 | 1.27 | 0.99 | < 0.001 | -0.47 | 0.51 | 0.12 | 0.28 |
| Rossano | Coastal | -4.89 | 1.21 | 0.99 | < 0.001 | 2.29 | 0.47 | 0.11 | 0.29 |
| Soveria | Inland | -6.84 | 1.13 | 0.99 | < 0.001 | 6.80 | 0.40 | 0.10 | 0.33 |
| Tropea | Coastal | 0.40 | 0.96 | 0.97 | < 0.001 | 9.54 | 0.36 | 0.08 | 0.37 |
| Vibo | Coastal | -0.92 | 1.03 | 0.98 | < 0.001 | 9.54 | 0.36 | 0.08 | 0.37 |

All linear correlations between WMO/GAW monitoring station temperature measurements and their ERA5 equivalents demonstrated strong agreement (all p-values < 0.001; R² ranging from 0.97 to 0.99), indicating a high degree of consistency between surface and ERA5 data. Conversely, no significant correlation was observed for RH (p-values between 0.28 and 0.37; R² ranging from 0.08 to 0.12), suggesting poor agreement between ground-based and ERA5 humidity data.

**Legend:** Results of the linear regression equations (int = intercept; m = slope) between monthly surface measurements of temperature (°C) and relative humidity (%) performed at the Lamezia Terme (LMT) WMO/GAW site, and their ERA5 counterparts.

**Figure S3: Relative humidity and events**

**Legend:** the figure shows temporal trends between relative humidity and monthly events over the year 2024.

**Table S6: Logistic regression analysis with the exact dates of outpatient visit or hospitalization due to symptomatic hyponatremia as dependent variable and each climatic variables as predictor.**

| **Climatic variable** | **Odds Ratio** | **2.5% - 97.5% CI** | **P value** | **q-value (after False Discovery Rate correction^§^)** |
| --- | --- | --- | --- | --- |
| Air daily temperature | 1.17 | 1.06 - 1.29 | 0.002 | 0.00840 |
| Air daily temperature SD | 1.36 | 0.87 - 2.13 | 0.182 | 0.38220 |
| Air daily minimum temperature | 1.12 | 1.03 - 1.22 | 0.01 | 0.03000 |
| Air daily maximum temperature | 1.19 | 1.08 - 1.32 | 0.0007 | 0.00840 |
| Air diurnal temperature range (DTR) | 1.15 | 0.98 - 1.36 | 0.09 | 0.21000 |
| Daily RH | 0.99 | 0.94 - 1.06 | 0.96 | 0.99000 |
| Daily RH SD | 0.99 | 0.87 - 1.13 | 0.93 | 0.99000 |
| Daily minimum RH | 0.99 | 0.95 - 1.03 | 0.62 | 0.86800 |
| Daily maximum RH | 0.98 | 0.92- 1.04 | 0.51 | 0.82385 |
| Diurnal RH range | 0.99 | 0.96 - 1.04 | 0.99 | 0.99000 |
| Roll-5T | 1.17 | 1.06- 1.29 | 0.002 | 0.00840 |
| Roll-5T max | 1.18 | 1.06 - 1.32 | 0.002 | 0.00840 |
| Roll-5T min | 1.16 | 1.05 – 1.28 | 0.003 | 0.01050 |
| Roll-5T range | 0.92 | 0.67 - 1.26 | 0.61 | 0.86800 |
| Roll-5RH | 0.99 | 0.91 – 1.09 | 0.88 | 0.99000 |
| Roll-5RH max | 0.96 | 0.88 - 1.05 | 0.38 | 0.66500 |
| Roll-5RH min | 1.02 | 0.94 – 1.10 | 0.67 | 0.87938 |
| Roll-5RH range | 0.96 | 0.90 – 1.03 | 0.28 | 0.53455 |
| Heath Index | 0.99 | 0.89- 1.12 | 0.99 | 0.99000 |
| Tropical nights | 2.72 | 1.02- 7.27 | 0.046 | 0.12075 |
| Heat waves | 4.87 | 1.75 - 13.5 | 0.002 | 0.00840 |

**Legend**: §= False Discovery Rate was performed according to Benjamini-Hochberg method.

**Table S7: Linear regression equations for temperature and relative humidity across the 10-day period preceding each event (from day -9 to day 0)**

| Dwelling place location | Date | Season | Temperature (°C) | | | | Relative Humidity (%) | | | |
| --- | --- | --- | --- | --- | --- | --- | --- | --- | --- | --- |
|  |  |  | int | m | R^2^ | *p*-value | int | m | R^2^ | *p*-value |
| Corigliano | Feb. 1^st^ | Winter | 14.6 | -0.11 | 0.04 | 0.602 | 59.2 | -0.11 | < 0.01 | 0.913 |
| Cinquefrondi | Mar. 29^th^ | Spring | 13 | 0.45 | 0.88 | < 0.001 | 74.3 | -1.48 | 0.39 | 0.055 |
| Davoli | Jun. 3^rd^ | Summer | 18.9 | 0.38 | 0.75 | 0.001 | 69.3 | -0.80 | 0.25 | 0.143 |
| Crotone | Jun. 4^th^ | Summer | 19.7 | 0.30 | 0.62 | 0.007 | 69 | -0.74 | 0.21 | 0.178 |
| Bonifati | Jun. 18^th^ | Summer | 25.4 | -0.32 | 0.38 | 0.057 | 57.7 | 0.95 | 0.21 | 0.186 |
| Marzi | Jun. 19^th^ | Summer | 23.9 | -0.05 | 0.01 | 0.736 | 63.9 | -0.05 | < 0.01 | 0.938 |
| Rende | Jul. 10^th^ | Summer | 25 | 0.08 | 0.05 | 0.55 | 65.1 | 0.55 | 0.24 | 0.148 |
| Vibo V. & Soveria M. | Jul. 15^th^ | Summer | 25.4 | 0.21 | 0.73 | 0.002 | 67 | 0.46 | 0.15 | 0.276 |
| Tropea | Jul. 22^nd^ | Summer | 27.1 | 0.14 | 0.54 | 0.016 | 71.7 | -0.68 | 0.42 | 0.042 |
| Isca | Jul. 29^th^ | Summer | 28 | -0.07 | 0.14 | 0.289 | 64 | 0.33 | 0.13 | 0.308 |
| Catanzaro | Aug. 1^st^ | Summer | 27.5 | 0.02 | < 0.01 | 0.823 | 68.2 | -0.53 | < 0.14 | 0.282 |
| Vibo | Aug. 3^rd^ | Summer | 27.2 | 0.06 | 0.08 | 0.441 | 68.8 | -0.87 | 0.31 | 0.095 |
| Rossano | Aug. 7^th^ | Summer | 28 | 0.01 | < 0.01 | 0.929 | 63.4 | 0.16 | < 0.01 | 0.784 |
| Rende | Sep. 23^rd^ | Fall | 19.5 | 0.30 | 0.63 | 0.006 | 57.1 | 1.13 | 0.42 | 0.043 |
| Rocca | Oct. 30^th^ | Fall | 21.7 | -0.26 | 0.86 | 0.24 | 77.4 | 0.53 | 0.47 | 0.519 |
| Gimigliano | Dec. 11^th^ | Winter | 14.5 | -0.32 | 0.54 | 0.015 | 80.2 | 0.50 | 0.04 | 0.6 |

**Legend:** results of the linear regression equations (int = intercept; m = slope) evaluating the behavior of environmental parameters on the day of each event, and the 9 days preceding it, for a total of 10 continuous days. Two events occurred in Vibo Valentia and Soveria Mannelli in the same day (July 15^th^).

**Table S8: Linear regression equations for 5-day moving averages of temperature and relative humidity across the 10-day period preceding each event (from day -9 to day 0)**

| Dwelling place location | Date | Season | Temperature (°C) | | | | Relative Humidity (%) | | | |
| --- | --- | --- | --- | --- | --- | --- | --- | --- | --- | --- |
|  |  |  | int | m | R^2^ | *p*-value | int | m | R^2^ | *p*-value |
| Corigliano | Feb. 1^st^ | Winter | 13.8 | -0.09 | 0.03 | 0.635 | 58.8 | 0.05 | < 0.01 | 0.883 |
| Cinquefrondi | Mar. 29^th^ | Spring | 13.5 | 0.37 | 0.98 | < 0.001 | 73.9 | -1.24 | 0.87 | < 0.001 |
| Davoli | Jun. 3^rd^ | Summer | 19 | 0.36 | 0.98 | < 0.001 | 69.5 | -0.77 | 0.78 | < 0.001 |
| Crotone | Jun. 4^th^ | Summer | 19.3 | 0.36 | 0.98 | < 0.001 | 68.8 | -0.71 | 0.71 | 0.002 |
| Bonifati | Jun. 18^th^ | Summer | 24.4 | -0.14 | 0.27 | 0.125 | 63.4 | -0.02 | < 0.01 | 0.887 |
| Marzi | Jun. 19^th^ | Summer | 23.8 | -0.03 | < 0.01 | 0.814 | 62.5 | 0.18 | 0.24 | 0.147 |
| Rende | Jul. 10^th^ | Summer | 24.7 | 0.14 | 0.43 | 0.041 | 66.1 | 0.38 | 0.44 | 0.036 |
| Vibo V. & Soveria M. | Jul. 15^th^ | Summer | 25 | 0.27 | 0.97 | < 0.001 | 68.1 | 0.31 | 0.29 | 0.107 |
| Tropea | Jul. 22^nd^ | Summer | 27.3 | 0.10 | 0.68 | 0.003 | 71.7 | -0.60 | 0.82 | < 0.001 |
| Isca | Jul. 29^th^ | Summer | 28.1 | -0.07 | 0.53 | 0.017 | 65.5 | 0.06 | 0.1 | 0.376 |
| Catanzaro | Aug. 1^st^ | Summer | 27.5 | 0.02 | 0.1 | 0.38 | 66.7 | -0.28 | 0.33 | 0.082 |
| Vibo | Aug. 3^rd^ | Summer | 27.4 | 0.05 | 0.54 | 0.016 | 67.9 | -0.65 | 0.73 | 0.002 |
| Rossano | Aug. 7^th^ | Summer | 27.5 | 0.07 | 0.59 | 0.009 | 62.9 | 0.20 | 0.07 | 0.454 |
| Rende | Sep. 23^rd^ | Fall | 20.6 | 0.13 | 0.45 | 0.034 | 59 | 0.92 | 0.89 | < 0.001 |
| Rocca | Oct. 30^th^ | Fall | NA | NA | NA | NA | NA | NA | NA | NA |
| Gimigliano | Dec. 11^th^ | Winter | 14.5 | -0.32 | 0.54 | 0.015 | 79.5 | 0.08 | < 0.01 | 0.897 |

**Legend:** results of the linear regression equations (int = intercept; m = slope) evaluating the behavior of environmental parameters on the day of each event, and the 9 days preceding it, for a total of 10 continuous days. Two events occurred in Vibo Valentia and Soveria Mannelli in the same day (July 15^th^). In this table, 5-day moving averages are considered.

**Table S9: Linear regression equations for maximum and minimum ranges of temperature and relative humidity across the 10-day period preceding each event (from day -9 to day 0)**

| Dwelling place location | Date | Season | Temperature (°C) | | | | Relative Humidity (%) | | | |
| --- | --- | --- | --- | --- | --- | --- | --- | --- | --- | --- |
|  |  |  | int | m | R^2^ | *p*-value | int | m | R^2^ | *p*-value |
| Corigliano | Feb. 1^st^ | Winter | 7.11 | -0.14 | 0.02 | 0.687 | 18.3 | 1.59 | 0.18 | 0.216 |
| Cinquefrondi | Mar. 29^th^ | Spring | 5.74 | 0.15 | 0.01 | 0.752 | 23 | -0.04 | < 0.01 | 0.98 |
| Davoli | Jun. 3^rd^ | Summer | 4.85 | 0.40 | 0.26 | 0.137 | 10.1 | 2.06 | 0.25 | 0.143 |
| Crotone | Jun. 4^th^ | Summer | 4.65 | 0.41 | 0.27 | 0.12 | 12.8 | 1.76 | 0.19 | 0.21 |
| Bonifati | Jun. 18^th^ | Summer | 8.76 | -0.29 | 0.05 | 0.531 | 39.2 | -2.51 | 0.42 | 0.042 |
| Marzi | Jun. 19^th^ | Summer | 3.58 | 0.54 | 0.46 | 0.031 | 24.4 | -0.33 | 0.04 | 0.584 |
| Rende | Jul. 10^th^ | Summer | 5.05 | 0.22 | 0.09 | 0.391 | 21.1 | -0.08 | < 0.01 | 0.951 |
| Vibo V. & Soveria M. | Jul. 15^th^ | Summer | 8.27 | -0.34 | 0.25 | 0.145 | 30.3 | -1.99 | 0.4 | 0.049 |
| Tropea | Jul. 22^nd^ | Summer | 4.41 | 0.32 | 0.12 | 0.326 | 9.2 | 1.64 | 0.26 | 0.131 |
| Isca | Jul. 29^th^ | Summer | 6.81 | -0.01 | < 0.01 | 0.977 | 24.6 | -0.89 | 0.08 | 0.442 |
| Catanzaro | Aug. 1^st^ | Summer | 5.24 | 0.20 | 0.08 | 0.439 | 14.1 | 0.75 | 0.09 | 0.413 |
| Vibo | Aug. 3^rd^ | Summer | 5.69 | 0.28 | 0.1 | 0.373 | 9.67 | 2.28 | 0.32 | 0.087 |
| Rossano | Aug. 7^th^ | Summer | 5.74 | 0.20 | 0.05 | 0.523 | 21 | 0.24 | < 0.01 | 0.877 |
| Rende | Sep. 23^rd^ | Fall | 7.49 | 0.11 | 0.12 | 0.316 | 30.1 | -0.01 | < 0.01 | 0.985 |
| Rocca | Oct. 30^th^ | Fall | NA | NA | NA | NA | 12.5 | 2.10 | 0.83 | 0.273 |
| Gimigliano | Dec. 11^th^ | Winter | 6.57 | 0.03 | < 0.01 | 0.897 | 33 | 0.29 | < 0.01 | 0.812 |

**Legend:** results of the linear regression equations (int = intercept; m = slope) evaluating the behavior of environmental parameters on the day of each event, and the 9 days preceding it, for a total of 10 continuous days. Two events occurred in Vibo Valentia and Soveria Mannelli in the same day (July 15^th^). In this table, ranges (max-min) are considered.
